# Transmission of extended spectrum β-lactamase-producing *Escherichia coli* and antimicrobial resistance gene flow across One Health compartments in eastern Africa: a whole-genome sequence analysis from a prospective cohort study

**DOI:** 10.1016/j.lanmic.2025.101224

**Published:** 2026-01

**Authors:** Patrick Musicha, Mathew A Beale, Derek Cocker, Fiona A Oruru, Allan Zuza, Chifundo Salifu, George Katende, Sylvia Nanono, Fred Isaasi, Kondwani Chidziwisano, Lawrence Mugisha, Henry Kajumbula, David Musoke, Tracy Morse, Shevin T Jacob, Nicholas A Feasey, Nicholas R Thomson

**Affiliations:** a Malawi Liverpool Wellcome Research Programme, Kamuzu University of Health Sciences, Blantyre, Malawi; b Department of Clinical Sciences, Liverpool School of Tropical Medicine, Liverpool, UK; c Wellcome Sanger Institute, Wellcome Genome Campus, Hinxton, UK; d Department of Pharmacology and Therapeutics, University of Liverpool, Liverpool, UK; e Infectious Disease Institute, Makerere University, Kampala, Uganda; f College of Health Sciences, Makerere University, Kampala, Uganda; g Malawi University of Business and Applied Sciences, Blantyre, Malawi; h College of Veterinary Medicine, Animal Resources and Biosecurity, Makerere University, Kampala, Uganda; i School of Public Health, Makerere University, Kampala, Uganda; j Department of Civil and Environmental Engineering, University of Strathclyde, Glasgow, UK; k Walimu, Kampala, Uganda; l School of Medicine, University of St Andrews, St Andrews, UK; m London School of Hygiene & Tropical Medicine, London, UK

## Abstract

**Background:**

The One Health paradigm considers interdependence of human, animal, and environmental health. However, there is little evidence from high-income countries to support the importance of a One Health approach to addressing spread of antimicrobial resistance (AMR). Given AMR is a global threat, understanding how the close interactions of humans with animals and the environment in low-income settings affect the spread of AMR is important. We aimed to investigate diversity and transmission of extended spectrum β-lactamase (ESBL)-producing *Escherichia coli* across household-linked One Health compartments using genomic data.

**Methods:**

We sequenced whole genomes of ESBL-producing *E coli* isolates from humans, animals, and the environment from a prospective, longitudinal cohort study conducted in Malawi (April 29, 2019, to Dec 3, 2020) and Uganda (July 16, 2020, to Aug 6, 2021). In the cohort study, 259 households were enrolled at baseline in Malawi and 92 in Uganda from a mix of urban, peri-urban, and rural areas. Households were followed up at months 1, 3, and 6 in Malawi and at months 1, 2, and 4 in Uganda. Samples collected at each visit included human and animal stool, environmental samples from hand-contact areas, food, and water, and broader environmental samples such as river water. Samples were cultured in buffered peptone water and then ESBL chromogenic agar to isolate ESBL-producing *E coli*. ESBL-producing *E coli* isolates underwent whole-genome sequencing. We performed phylogenetic analyses, and in-silico multi-locus sequence typing, characterised AMR determinants and linked genotypes with sample location, ecological source, and other covariates. We performed fine-scale single nucleotide polymorphism (SNP) and network analysis to infer strain and plasmid transmission across ecological compartments. The primary outcome was colonisation with ESBL-producing *E coli*. Secondary outcomes were genomic clusters and ESBL genomic determinants within and between One Health compartments.

**Findings:**

We found high diversity of ESBL-producing *E coli*, with 170 sequence types and 166 genomic clusters identified from 2344 genomes, including 1814 genomes from Malawi (907 human, 221 animal, and 686 environmental) and 530 genomes from Uganda (380 human, 147 animal, and three environmental). Sequence type (ST)131 dominated in Malawi (209 [11·5%] of 1814 genomes), and ST10 dominated in Uganda (45 [8·5%] of 530 genomes). Common ESBL genes *bla*_CTX−M−15_ (1604 [68·4%] of 2344 genomes) and *bla*_CTX−M−27_ (336 [14·3%] of 2344 genomes) were carried on a complex network of 55 and 30 different plasmids. This diversity of plasmids presented multiple pathways for dissemination and revealed high force of selection. Phylogenetic analyses revealed common intermixing of isolates between humans, animals, and the environment. SNP transmission analysis revealed ecologically overlapping clusters, suggesting ESBL-producing *E coli* co-circulation both within and between compartments with frequent spillover events. Applying a five-SNP threshold, we inferred 463 human–environment transmission events, 146 human–animal events, and 142 animal–environment events.

**Interpretation:**

Our work suggests that a One Health approach is crucial to addressing AMR in eastern Africa. Improving water, sanitation, and hygiene systems will create a safer environment, reduce spillovers of AMR bacteria between compartments, and eventually reduce AMR reservoirs in the environment and in animals.

**Funding:**

Medical Research Council, National Institute for Health and Care Research, and Wellcome Trust.

## Introduction

*Escherichia coli* is found ubiquitously as a commensal in the intestinal tracts of humans and warm-blooded animals but can also cause a range of diseases. In humans, these diseases include both diarrhoeal and extra-intestinal infections (eg, urinary tract and bloodstream infections)^1^ that can be life-threatening, especially in clinically vulnerable populations including neonates and immune-compromised individuals.^2^ The threat to human health posed by *E coli* is exacerbated by the spread of antimicrobial resistance (AMR). AMR is estimated to have caused 1·14 million human deaths in 2021, and *E coli* was identified among the leading aetiological agents of deaths attributable to AMR.^3^ Of major concern are extended spectrum β-lactamase (ESBL)-producing *E coli,* which are resistant to third-generation cephalosporins and classified by WHO as pathogens of critical priority.^4^ In the past two decades, *E coli* resistant to third-generation cephalosporins has become more common in all world regions including Africa.^3^^,^^5^ Although evidence on the burden of third-generation cephalosporin-resistant *E coli* infections in Africa is sparse, there are growing indications that they cause higher mortality and morbidity risk than third-generation cephalosporin-susceptible *E coli* infections.^6^^,^^7^

Discerning the full diversity of AMR bacteria across different ecological niches and geographical settings is important for identifying reservoirs and sources of AMR and how AMR bacteria are transmitted between those ecological niches and settings. However, our understanding of ESBL-producing *E coli* diversity is not only limited to studies biased towards samples from human and health-care settings, but also does not have comprehensive geographical and temporal representation. The One Health framework proposes a holistic approach to designing and implementing interventions for combating health threats. Although this approach is viewed as particularly important for AMR, the evidence to support it is sparse.^8^^,^^9^ Studies from the UK and Italy found little evidence of relatedness and transmission of ESBL-producing *E coli* and carbapenemase-producing *Klebsiella pneumoniae* between humans and animal or environmental sources.^10^^,^^11^ However, studies from Africa and other low-income and middle-income locations have shown close genomic relatedness of isolates across these ecological compartments, albeit with limited data.^12^, ^13^, ^14^ These studies both pose questions around the degree to which controlling the spread of AMR truly requires a One Health solution and highlight the pressing need to test the generality of their observations in multiple other environments by conducting research with targeted genotypic datasets across veterinary and public health sectors, especially in countries with different agricultural, political, and sociological conditions.

The Drivers of Antimicrobial Resistance in Uganda and Malawi (DRUM) project took a One Health approach to understand how water, sanitation, and hygiene (WASH) practices interact with antimicrobial usage to facilitate transmission of ESBL-producing Enterobacteriaceae in Uganda and Malawi. Here, our study aimed to investigate the genomic diversity and transmission of ESBL-producing *E coli* across household-linked One Health compartments in the two coutries.

## Methods

### Study design and participants

The DRUM household prospective cohort study took place from April 29, 2019, to Dec 3, 2020, in Malawi (Chileka, Chikwawa, and Ndirande) and from July 16, 2020, to Aug 6, 2021, in Uganda (Kampala and Hoima). 259 households were enrolled at baseline and followed up at months 1, 3, and 6 in Malawi, and 92 households were enrolled at baseline and followed up at months 1, 2, and 4 in Uganda. Household selection and participant enrolment have previously been described in the protocol.^15^ Briefly, sites were selected in Malawi to represent urban, peri-urban, and rural settings; and on the basis of community acceptability and existing research infrastructure. A comparable mixture of sites was selected in Uganda to include diverse socioeconomic settings.^15^

The study was approved by the College of Medicine Research Ethics Committee in Malawi (P.11/18/2541); Makerere University School of Veterinary Medicine and Animal Resources (SVARREC/18/2018), Joint Clinical Research Centre (JC3818), and Uganda National Council for Science and Technology (UNCST; HS489ES) in Uganda; and Liverpool School of Tropical Medicine Research and Ethics Committee (18-090). Informed written consent was obtained from all participants in their local language. DNA transfer was compliant with the Nagoya Protocol on Access and Benefits Sharing (export permit MEPA-12-07-1313-21-16c in Malawi and waived by the UNCST in Uganda as research intended for educational purposes).

### Procedures

At each household visit, human and animal stool samples were collected alongside household environmental samples from hand-contact areas, food, water, and broader environmental samples including river water. Samples were initially cultured in buffered peptone water and then ESBL chromogenic agar (CHROMagar, Saint-Denis, France) to isolate ESBL-producing *E coli* (appendix 1 p 2).

Libraries for genomic DNA of all ESBL-producing *E coli* isolates were constructed using NEB Ultra II custom kit on Agilent Bravo WS automation system and sequenced on the Illumina HiSeq X10 platform (Illumina, San Diego, CA, USA) at the Wellcome Sanger Institute, UK. Raw reads were assembled into contiguous sequences (contigs) using Spades (version 3.14.0) and annotated using PROKKA (version 1.14.5).^16^^,^^17^ We performed pangenome analysis and generated a core gene alignment using panaroo (version 1.3.0).^18^ A core gene maximum likelihood phylogenetic tree was constructed using IQ-TREE (version 1.6.12) with GTR+I+G model.^19^ We performed population structure analysis on the whole collection using PopPUNK (version 2.6) and assigned lineages based on a previously described PopPUNK reference database for *E coli.*^20^^,^^21^ Multi-locus sequence typing (MLST) was performed in silico with the mlst tool (version 2.16.2) to determine sequence types. We screened for AMR determinants using AMRFinderPlus (version 3.10.40) and used MOB-suite (version 3.0.3) to cluster and characterise plasmid contigs.^22^^,^^23^ We analysed the genomes from the two countries together rather than separately to investigate how bacteria or AMR genes stratified across the countries, and whether transmission patterns observed were generalisable across the countries.

For each major sequence type (with ≥100 genomes), we randomly selected two to six isolates for long-read sequencing on Sequel II (PacBio, Menlo Park, CA, USA). We combined long (PacBio) and short (Illumina) reads to construct high-quality hybrid reference genomes for each of the major sequence types. Illumina raw reads for all other genome sequences were then mapped to one of the generated reference genomes to call single nucleotide polymorphisms (SNPs) and accurately generate sequence type-specific alignments. We inferred putative transmission networks by constructing edge-networks from all pairwise comparisons below a series of thresholds (zero, one, two, five, ten, and 20 SNPs). We quantified putative transmission events at each threshold by counting the number of pairwise interactions between and within each compartment after deduplication. Bioinformatics analyses are described in more detail in appendix 1 (pp 3–5).

### Outcomes

The primary outcome was colonisation with ESBL-producing *E coli*. Secondary outcomes were genomic clusters and ESBL genomic determinants within and between One Health compartments.

### Statistical analysis

Sample sizes were not determined a priori due to the large number of variables and potential unknown parameters; to ensure representation of study populations, households were sampled using an inhibitory with close pairs design extended to allow for sampling within sites with spatially heterogeneous populations.^24^^,^^25^ Associations between sequence type versus country and sequence type versus ecological compartment were performed using χ^2^ test or Fisher’s exact test, as appropriate. We performed a multinomial regression to quantify association between plasmid cluster as outcome and sample country of origin and ecologic source (appendix 1 pp 5–6). All statistical analyses were performed in R version 4.3.1.

### Role of the funding source

The funders of the study had no role in study design, data collection, data analysis, data interpretation, or writing of the report.

## Results

We sequenced 2828 isolate genomes identified as likely ESBL-producing *E coli*, of which 2252 (79·6%) were from Malawi and 576 (20·4%) were from Uganda. After performing quality filtering on raw Illumina reads and genome assemblies, 2344 (82·9%) of 2828 sequenced genomes were identified as high-quality *E coli* genomes, including 144 *Shigella* spp. Here, we treated the *Shigella* spp as a specialised *E coli* pathovar and included them in the analysis due to their phylogenetic indistinguishability from *E coli*. 1814 (77·4%) of 2344 genomes came from Malawi, of which 907 (50·0%) were from human stool, 221 (12·2%) were from animal stool, and 686 (37·8%) were from the environment. 530 (22·6%) of 2344 genomes came from Uganda, of which 380 (71·7%) were from human stool, 147 (27·7%) were from animal stool, and three (0·6%) were from the environment.

**Figure 1:**
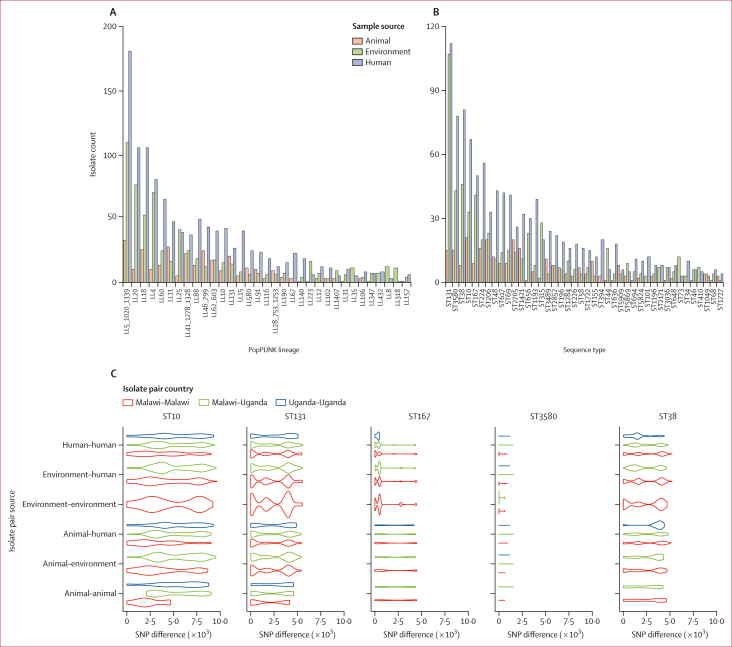
Diversity of extended spectrum β-lactamase-producing *Escherichia coli* isolates from Uganda and Malawi Analysis at the population level (genomic clusters defined using both PopPUNK and multi-locus sequence types) indicates a large number of genomic lineages (A) and sequence types (B), in both cases the majority of which are shared by all ecological niches. Panels A and B include PopPUNK lineages and sequence types with a frequency of at least ten genomes. (C) Analysis of pairwise SNP distributions within sequence types shows both close (including identical genomes) and distant genomic relationships. SNP=single nucleotide polymorphism. ST=sequence type.

The pan-genome for the 2344 isolates comprised 23 677 genes, of which 3066 (12·9%) were identified in at least 99% of the genomes and, thus, considered to be core. We inferred a phylogeny from 268 606 SNP sites within the core gene alignment showing the ESBL-producing *E coli* from the DRUM study to be highly diverse, with multiple phylogenetic clusters including genomes of isolates from both Malawi and Uganda, and from the three ecological compartments we sampled from (appendix 1 p 7). The genomes were assigned to 166 PopPUNK lineages, but despite apparent high lineage diversity, the distribution of genomes was highly skewed, with 1675 (71·5%) of 2344 isolate genomes belonging to only 16 (9·6%) of 166 ESBL-producing *E coli* lineages. These 16 high-frequency lineages comprised between 40 and 313 isolate genomes each (figure 1A). Conversely, most of the lineages (110 [66·3%] of 166) were uncommon, containing fewer than five genomes each.

The high diversity was replicated by MLST (figure 1B), whereby 2239 (95·5%) of 2344 ESBL-producing *E coli* genomes were typed into 170 known sequence types. 85 (50·0%) of 170 known sequence types were either singletons or contained just two genomes. Sequence type (ST)131 was the most common (234 [10·0%] of 2344 genomes), followed by ST3580 (136 [5·8%]), ST38 (135 [5·8%]), ST10 (121 [5·2%]), and ST167 (100 [4·3%]; figure 1). Although the most common sequence types and PopPUNK lineages included isolates from both Malawi and Uganda, there were significant differences in sequence type distribution between the two countries (Fisher’s exact test simulated p<0·0001), with ST131 (209 [11·5%] of 1814 genomes) predominating in Malawi and ST10 (45 [8·5%] of 530 genomes) predominating in Uganda (appendix 1 p 8). 27 sequence types were *Shigella* spp specific, the most common being ST59 (59 [41·0%] of 144 genomes) and ST46 (14 [9·7%]), both specific to *Shigella dysenteriae*. ST3580 was the major sequence type, which had genomes for both *E coli sensu stricto* and *Shigella sonnei*. We also found that 105 (4·5%) of 2344 genomes were not present in existing MLST databases; these genomes were submitted to Enterobase.^26^ Notably, the majority of new sequence types were related to the ST10 (28 [26·7%] of 105 genomes), ST155 (19 [18·1%]), and ST131 (7 [6·7%]) clonal complexes, indicating they are closer relatives of these dominant sequence types (appendix 1 pp 14–15).

**Figure 2:**
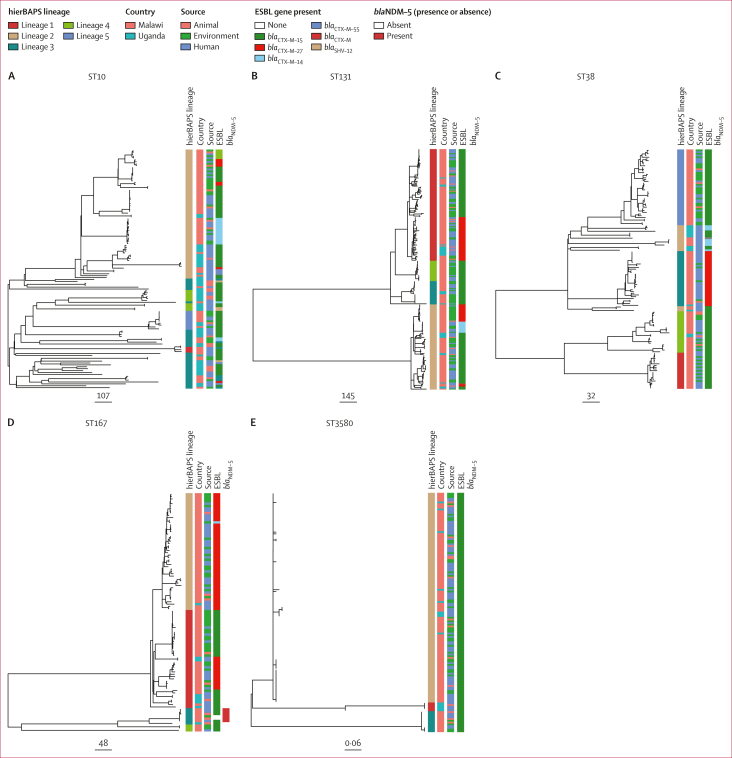
Phylogenetic analysis of common ESBL-producing sequence types in Malawi and Uganda Maximum likelihood phylogenetic trees of ST10 (A), ST131 (B), ST38 (C), ST167 (D), and ST3580 (E) genomes (all n≥100) from the DRUM collection. Mapped to trees are level 1 hierBAPS lineages, isolate country of origin, sample ecological source, type of ESBL gene present in a genome, and presence or absence of the carbapenemase *bla*_NDM-5_ gene. ESBL=extended spectrum β-lactamase. hierBAPS=hierarchical Bayesian analysis of population structure. ST=sequence type.

Within individual sequence types, we examined number of core gene SNPs and found this varied, with some sequence types showing low diversity (all genomes separated by no more than five core SNPs), while other sequence types had high diversity (some genomes separated by more than 1·0 × 10^4^ core SNPs; figure 1C; appendix 1 p 9). Due to these wide variations, sequence types provided insufficient resolution for investigating relative diversity and potential transmission between different ecological niches and settings. Across the five major sequence types (with ≥100 genomes), phylogenetic and population structure analyses revealed strong intermixing of isolates from all three ecological compartments; although interspersing of isolates from the two countries could be observed, we also found phylogenetic clustering of genomes by country of origin (figure 2, appendix 1 p 6).

**Figure 3:**
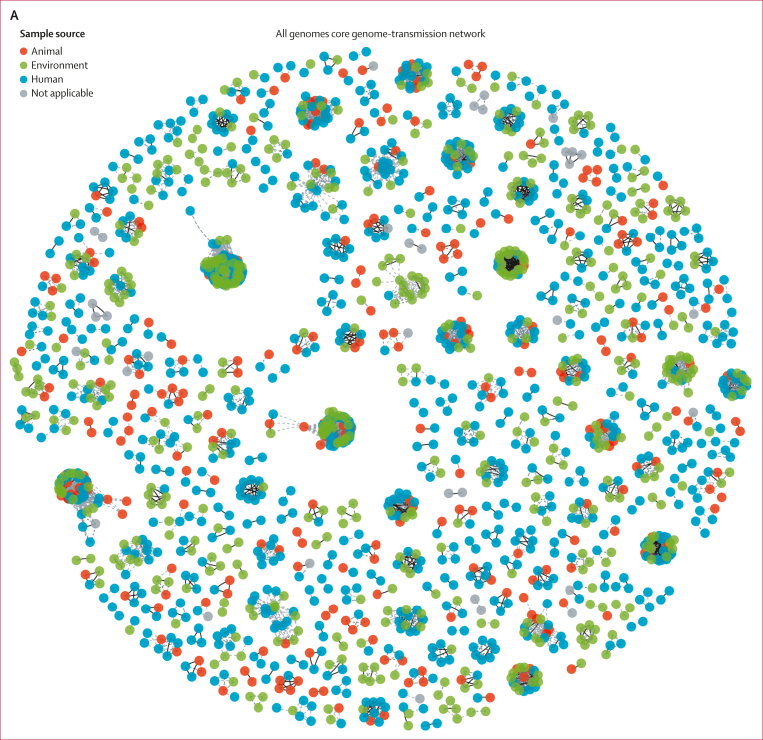

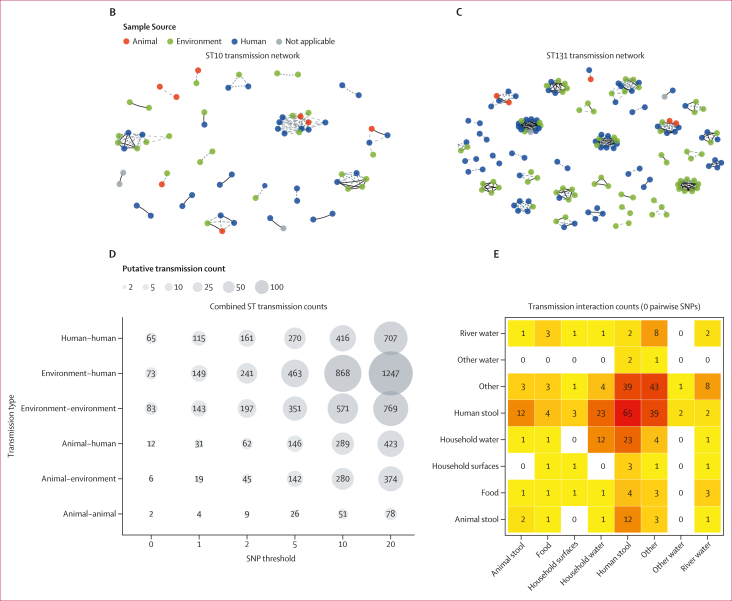
Genomic relationships between samples from ecological compartments (A) Core genome pairwise SNP distance-based transmission network for all extended spectrum β-lactamase-producing *Escherichia coli* genomes in the DRUM collection with ≤5 pairwise SNP distances. Nodes are coloured by source, and edges indicate samples are connected by ≤5 SNPs. Nodes coloured in grey indicate missing data. Lineage-specific reference mapped analysis of within and between ecological niche transmission networks of ST10 (B) and ST131 (C) genomes with ≤5 SNP distances. (D–E) Counts of transmission events between and within ecological compartments at SNP thresholds of zero, one, two, five, ten, and 20. For panel E, environmental compartment has been split into specific sources of the environmental samples. SNP=single nucleotide polymorphism. ST=sequence type.

At different SNP thresholds of zero, one, two, five, ten, and 20, we found evidence of both within-niche and between-niche transmission using both the core gene network (figure 3A) and the sequence type-specific networks (figure 3B and C). Notably, we found that despite the human compartment being the most sampled (followed by the environment), human-to-human transmission events were consistently fewer than environment–human transmission events (after deduplication) across all the SNP thresholds; figure 3D). At the five-SNP threshold, we inferred 463 transmission events between humans and the environment, 146 events between humans and animals, and 142 events between animals and the environment. Separating the environmental isolates into specific specimen types (appendix 2), we showed that at the highest level of genomic similarity (zero-SNP threshold), the most frequent between-niche transmission events were those between human and household water and human and animal stool (figure 3E). By comparison, food represented only a small part of inferred transmissions. We overlayed household data to the core SNP network and discovered that isolates from the same household clustered together (appendix 1 p 10). However, most core-SNP network clusters contained isolates from a mixture of households (appendix 1 p 10), providing evidence for how within-household and between-household direct transmission is contributing to the widespread dissemination of ESBL-producing *E coli* across communities.

**Figure 4:**
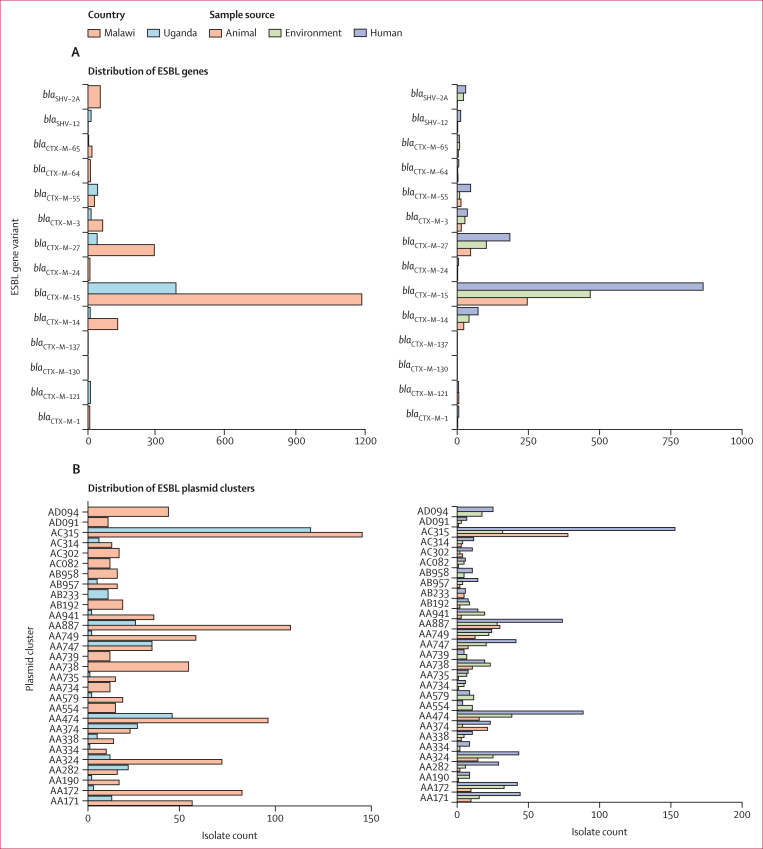

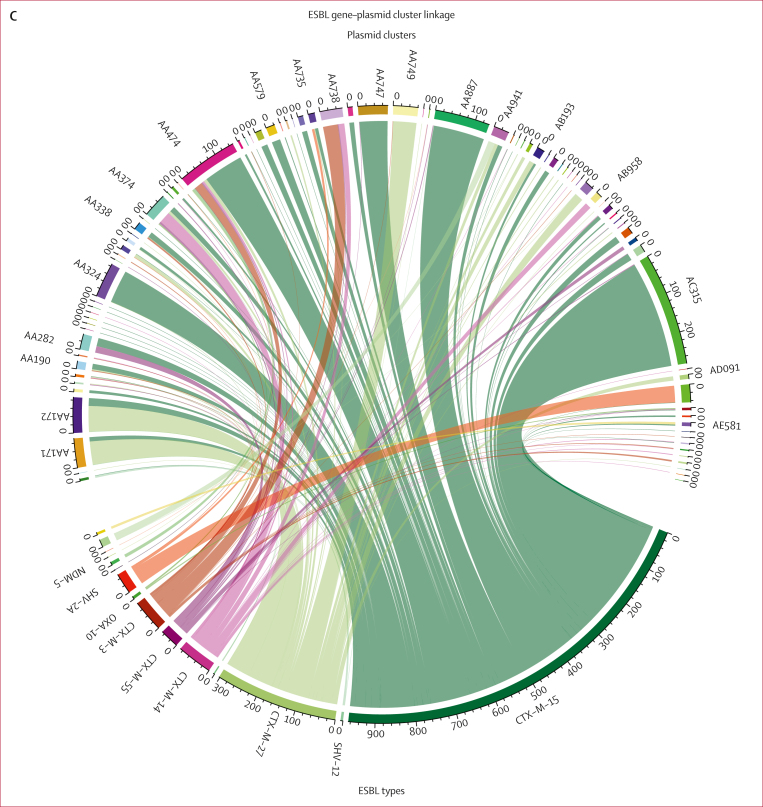
ESBL genes and plasmids diversity and overlap across ecological compartments and countries (A) Distribution of ESBL gene variants by country and ecological compartment. (B) Distribution of ESBL gene-carrying plasmid clusters by country and ecological compartment. (C) Circos plot showing links between plasmids clusters (top tracks) and the ESBL type (bottom track) encoded by the gene encoded on the plasmid. ESBL=extended spectrum β-lactamase.

We identified 150 unique AMR-associated genes and point mutations. Most of these genes or mutations were present in isolates from all three ecological compartments (human stool, animal stool, and the environment; appendix 1 p 11). AMR genes included 20 ESBL genes, of which *bla*_CTX–M–15_ (1604 [68·4%] of 2344 genomes) was the most common, followed by *bla*_CTX−M−27_ (336 [14·3%] of 2344 genomes) and *bla*_CTX−M−14_ (143 [6·1%] of 2344 genomes), with other ESBL genes less frequently distributed across genomes (figure 4A). The distribution of ESBL genes varied across different sequence types, with some sequence types such as ST10 and ST131 (figure 2A–D) having more than one ESBL gene, whereas isolates in sequence types such as ST3580 (figure 2E) carried only one ESBL gene. When more than one ESBL gene was present in a single sequence type, there was evidence of genes being linked to specific phylogenetic clusters (figure 2A–E). Among *Shigella* spp isolates, the distribution of the ESBL genes was similar to the overall collection, with *bla*_CTX−M−15_ being the dominant ESBL gene (121 [84·0%] of 144 genomes), followed by *bla*_CTX−M−3_ (nine [6·3%] of 144 genomes) and *bla*_CTX−M−27_ (seven [4·9%] of 144 genomes).

We also identified the carbapenamase gene *bla*_NDM−5_ in six genomes and two colistin resistance associated mutations: *pmrB* E123D in 343 (14·6%) of 2344 genomes and *pmrB* Y358N in 674 (28·8%) of 2344 genomes (appendix 1 p 11). The chromosomal mutations *pmrB* E123D and *pmrB* Y358N were spread across multiple lineages with isolates from all the three ecological niches (appendix 1 p 11). However, the six genomes carrying the *bla*_NDM−5_ all belonged to isolates from a distinct genome cluster of ST167, of which three were from human, two from animal, and one from environmental sources (figure 2D, appendix 1 p 11). These six isolates came from one household, suggesting a common source.

Given plasmids are one of the primary vectors of AMR genes, we reconstructed and clustered individual plasmid sequences from our genome assemblies using MOB-suite to understand their diversity across compartments in this setting and their role in the distribution of AMR genes, with a focus on ESBLs. This analysis revealed a high diversity of plasmids shared across compartments, with 595 distinct plasmid clusters overall, of which 193 (32·4%) carried AMR-associated genes and 89 (15·0%) carried ESBL genes. Figure 4C shows the importance of studying both AMR-associated genes and vectors independently: individual ESBL genes were widely distributed across multiple plasmid clusters (eg, *bla*_CTX−M−15_ was found in 55 plasmid clusters, *bla*_CTX−M−27_ in 30 plasmid clusters, and *bla*_CTX−M−14_ in 21 plasmid clusters). Conversely, the majority of plasmid clusters were linked to one (48 [53·9%] of 89 plasmid clusters) or two (22 [24·7%]) ESBL genes. This finding includes the dominant plasmid clusters found in both countries (plasmid clusters AC315, AA324, and AA887), which were linked exclusively to *bla*_CTX−M−15_ (figure 4C). This observation was also true for other (non-ESBL) AMR genes (appendix 1 p 12). The maximum number of distinct ESBL genes seen in an individual plasmid cluster was eight (cluster AA474). Fitting a fixed-effects multinomial regression to the plasmid cluster data with country and ecological source as predictor variables, we found that most plasmid clusters were not associated with a particular country, exceptions being four plasmid clusters less likely to be identified in isolates from Uganda relative to Malawi (AA172, AA738, AA749, and AD094) and five plasmid clusters more likely to be identified in isolates from Uganda than Malawi (AA282, AA347, A474, AA747, and AC315; appendix 1 p 13). In addition, cluster AA747 was more likely to be identified in environmental isolates than in animal or human isolates, AD094 was more likely to be identified with environmental and human samples than animal isolates, and AA374 was more likely in animal isolates than humans or the environment (appendix 1 p 13). Across the *Shigella* spp genomes, the distribution of major plasmid clusters mirrored that of the overall collection, with AC315 and AA887, the dominant plasmid clusters in the overall collection, also being the most common among the *Shigella* spp genomes.

**Figure 5:**
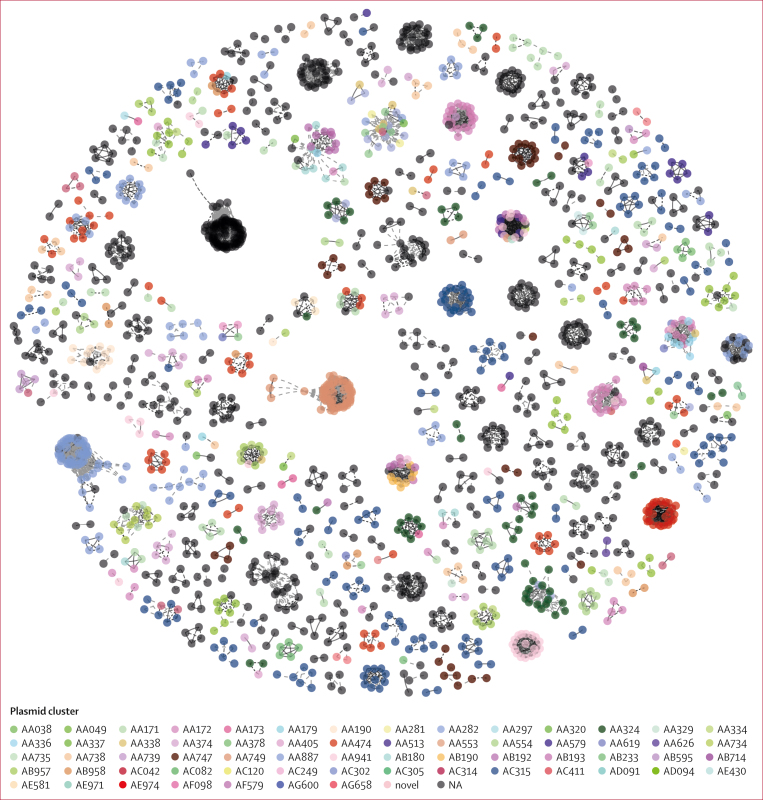
Distribution of ESBL plasmid clusters across ESBL *Escherichia coli* transmission clusters Core genome pairwise SNP distance-based transmission network for all ESBL-producing *Escherichia coli* genomes in the DRUM collection with ≤5 pairwise SNP distances. Nodes represent genomes coloured by ESBL-plasmid cluster (pale grey genome nodes do not have an ESBL plasmid), and edges indicate sample connections by ≤5 SNPs. Clusters of linked genomes form putative transmission networks. ESBL=extended spectrum β-lactamase. NA=not applicable. SNP=single nucleotide polymorphism.

Finally, we overlayed the plasmid cluster data to the core-SNP bacterial host transmission network (figure 5). Although there were core-SNP clusters with a mixture of plasmids, isolates in most core-SNP network clusters shared the same ESBL plasmid, meaning that the isolates in these clusters were not only similar at chromosomal level but also shared the same ESBL plasmids. However, some of the common plasmid clusters—such as AC315, AA474, and AA887—were shared across multiple bacterial host core-SNP clusters (figure 5). We also identified a substantial number of clusters comprising genomes with ESBL genes but not linked to any plasmid cluster. Genomes not linked to any ESBL plasmid included 639 (39·8%) of 1604 genomes carrying *bla*_CTX−M−15_, which is known to be both plasmid-borne and chromosome-borne. We investigated the genomic environments of the ESBL genes for ten randomly selected genomes and found that in all of those genomes, the ESBL genes were on a chromosome (appendix 1 p 14).

## Discussion

In this study, we investigated the genomic diversity and transmission of ESBL-producing *E coli* in Malawi and Uganda using prospective, longitudinally collected samples from humans, animals, and the environment.^15^^,^^27^ All samples were epidemiologically linked at the household level, or in the case of river water, at the level of the geographical area in which the study was being conducted.^14^ The direct epidemiological link between genetic diversity to ecological source enabled us to unravel ongoing transmission between these ecological compartments, both within and between households. Here, we showed that ESBL-producing *E coli* were highly diverse. This high diversity was observed at both the strain level and at the level of the ESBL genetic determinants (including both the ESBL genes themselves, and the plasmids that harbour them).

We also found multiple clusters of closely related ESBL-producing *E coli* strains and ESBL determinants that, crucially, were shared by different ecological compartments, indicating frequent sharing or transmission of ESBL-producing *E coli* or ESBL determinants between these compartments. Although there were differences in distribution of isolates by lineage between Malawi and Uganda, most sequence types and all the ESBL genes and plasmids were found in both countries, indicating that the transmission of these strains and the ESBL gene flow in the two countries are independently driven by common selection pressures, most likely from widespread use of third-generation cephalosporins in both countries. However, we were unable to find out whether there was transmission between the countries with the available data.

Globally, ESBL production in *E coli* has been associated with a small number of sequence types and AMR genes, with ST131 and *bla*_CTX−M−15_ being the most prominent.^28^ This observation is true for Malawi, where ST131 and *bla*_CTX−M−15_ have previously been identified as the leading ESBL sequence type and gene, respectively.^29^^,^^30^ However, this study has captured far more strain and ESBL gene diversity than the previous studies. For Uganda, we found a contrasting genomic epidemiology of ESBL-producing *E coli*, in that ST10 rather than ST131 was the dominant ESBL-producing *E coli* sequence type. The high diversity of ESBL-producing *E coli* that this study captured in both Malawi and Uganda shows the widespread dissemination of ESBL determinants, especially *bla*_CTXM−15_, to lineages they were not previously observed in, such as the novel sequence types identified through Enterobase (appendix 1 pp 14–15). Although the high burden of bacterial infections and subsequent high usage of third-generation cephalosporins in sub-Saharan Africa should be expected to drive the acquisition and spread of ESBL-producing *E coli*, the sharing of plasmids by distinct multiple lineages and transmission clusters means that even when transmission cannot occur at a bacterial host strain level, plasmids facilitate horizontal transfer of ESBL genes across lineages and of the genes between plasmid vectors. Given the nature and probable frequency of exposure and transmission we have revealed in this setting, our data describe a truly One Health view of AMR at the level of the bacterial host, the plasmid vector, and the individual AMR gene and across ecological compartments.^28^

Our study underlines the need to use genomic data to understand reservoirs and routes of AMR transmission in different geographical and socioeconomic contexts to develop effective interventions against AMR transmission. It provides a unique view of AMR in this setting, revealing the multiple pathways for successful dissemination of ESBL genes. More specifically, the reservoirs of AMR genes might not necessarily be clinical nor human, and the One Health framework provides a holistic approach for addressing the AMR problem.^8^ Here, the One Health framework informed our sampling strategy, enabling us to show that highly similar ESBL-producing *E coli* strains and ESBL determinants are often shared between humans, animals, and the environment in these African settings. The overlap of genomic clusters observed in this study has been observed elsewhere in Africa, but, just as is the case between Malawi and Uganda, with different sequence type distributions.^12^^,^^13^ Our findings and those from the other African studies contrast markedly with findings from high-income countries with robust sanitation systems, which suggest little transmission of AMR bacteria or flow of genetic determinants of AMR between different ecological compartments.^10^ In settings such as those of our study, human interactions with animals and the environment are fundamentally different to those of many high-income settings. It is common for domestic livestock animals (eg, poultry and goats) and companion animals (eg, dogs and cats) to share homes with humans.^27^ Specifically, we have identified household stored water to be a major source or reservoir of ESBL-producing *E coli* shared with humans. This observation could be linked to poor WASH practices and infrastructure, which lead to high amounts of enteric bacteria being shed into the environment through human and animal faeces. High frequency of contact between humans, animals, and the environment probably facilitates increased transmission of ESBL-producing *E coli* and other enteric bacteria between the different ecological sources. However, future studies should investigate directionality of transmission to identify primary sources and, in a setting where universal access to clean water is unlikely to be achieved soon, evaluate non-WASH interventions that would be effective in interrupting community AMR transmission.

Study limitations include 17% of genomes being excluded at quality control, few environmental samples from Uganda, and more samples from Malawi than Uganda in general. Apart from Malawi having more study sites, sample collection was more heavily affected by the COVID-19 pandemic in Uganda, which had a far stricter lockdown, limiting importation of research consumables and participant recruitment. This limitation ultimately affected the richness of the data from Uganda, but not the analysis. Therefore, many of our conclusions are based on the Malawi data, but the findings are broadly corroborated by data from Uganda. Additionally, our plasmid clustering approach using MOB-suite was dependent on the plasmid reference database, hence novel plasmids could have been missed. Our dataset offers opportunities to investigate ESBL-producing *E coli* diversity and transmission in relation to other variables such as rural versus urban; however, to do this satisfactorily, additional data such as differences in access to antimicrobials, demographic information, and population mobility would be needed.

By taking a One Health approach to the study of ESBL-producing *E coli,* we reveal that, in eastern Africa, genetically similar ESBL-producing *E coli* strains and ESBL determinants are shared and transmitted between all relevant ecological compartments at the individual level and the household level. The success of different *E coli* sequence types, the mobility of ESBL genes via multiple plasmid vectors and the ability of plasmids to successfully disseminate these genes into new lineages provide multiple pathways for the successful dissemination of ESBL genes across households and between country states in the region. Using ESBL-producing *E coli* as an exemplar, we provide the high-resolution data showing AMR is indeed a One Health problem in an African context.

## Data sharing

Raw sequence data were deposited in the European Nucleotide Archive (https://www.ebi.ac.uk/ena). Genome sample accession numbers and associated metadata are included in appendix 2. The study protocol is published elsewhere.^15^

## Declaration of interests

NAF received salary funding from a National Institute for Health and Care Research Global Health Professorship via Liverpool School of Tropical Medicine and University of St Andrews. All other authors declare no competing interests.

## Supplementary Material

Supplementary appendix 2

Research in context Evidence before this study We searched PubMed for studies in all languages, published from database inception to Oct 6, 2024, investigating transmission or relatedness of drug-resistant or extended spectrum β-lactamase (ESBL)-producing *Escherichia coli* in Africa. We used the search terms: (“E coli” OR “*Escherichia coli*” OR “Enterobacteriaceae” OR “Enterobacteriales”) AND (“resistan∗” OR “ESBL” OR “Extended spectrum beta-lactamase”) AND (“Diversity” OR “Transmission” OR “relatedness”) AND (“genom∗” OR “phylog∗”) AND ((“Angola” OR “Benin” OR “Botswana” OR “Burkina Faso” OR “Burundi” OR “Cameroon” OR “Cape Verde” OR “Central African Republic” OR “Chad” OR “Comoros” OR “Republic of the Congo” OR “Congo Brazzaville” OR “Democratic Republic of the Congo” OR “DRC” OR “Cote d’Ivoire” OR “Djibouti” OR “Equatorial Guinea” OR “Eritrea” OR “Ethiopia” OR “Gabon” OR “The Gambia” OR “Ghana” OR “Guinea” OR “Guinea-Bissau” OR “Ivory Coast” OR “Kenya” OR “Lesotho” OR “Liberia” OR “Madagascar” OR “Malawi” OR “Mali” OR “Mauritania” OR “Mauritius” OR “Mozambique” OR “Namibia” OR “Niger” OR “Nigeria” OR “Réunion” OR “Rwanda” OR “São Tomé and Principe” OR “Senegal” OR “Seychelles” OR “Sierra Leone” OR “Somalia” OR “South Africa” OR “Sudan” OR “South Sudan” OR “Swaziland” OR “Eswatini” OR “Tanzania” OR “Togo” OR “Uganda” OR “Western Sahara” OR “Zambia” OR “Zimbabwe”) OR “Africa”)). After reviewing titles and abstracts of identified publications, we identified ten relevant articles. Except for one article with 816 genomes, most of the articles included small numbers of genomes (range 12–169) and used low-resolution data, including multi-locus sequence types and antimicrobial resistance (AMR) gene profiles, to identify similarity of isolates or transmission across different ecological compartments. Added value of this study By using one of the largest collections of epidemiologically linked *E coli* isolates from different ecological compartments in sub-Saharan Africa to date and by exploiting the resolution provided by whole-genome sequencing, our study provides novel insights into AMR transmission between humans, animals, and the environment in eastern Africa. Results of our high-resolution genomic analysis highlight a high number of putative transmission events between humans, animals, and the environment and the sharing of ESBL determinants, supporting the view that AMR is a One Health problem in eastern African contexts. Implications of all the available evidence The findings from this study are consistent with similar studies from other settings within the African context. In contrast to findings in studies from high-income settings, these African studies emphasise the importance of applying a One Health approach to address AMR in low-income settings, where human, animal, and environmental interactions might facilitate the spread of drug-resistant bacteria. This spread is probably a consequence of inadequate water, sanitation, and hygiene infrastructure, contributing to an unsafe environment. The identification of multiple pathways of ESBL *E coli* transmission, including both direct strain transmission and horizontal gene transfer, underscores the complexity of AMR dissemination. Therefore, our work highlights that data from surveillance of AMR bacteria can be used as a marker of system failure; for example, in this case, detection of AMR enteric bacteria indicates inadequacies of sanitation infrastructure. This work contributes valuable data to the global genomic repository of AMR bacteria and underscores the need for more comprehensive AMR surveillance globally and for interventions tailored to the specific ecological and socioeconomic contexts of low-income settings.
