# Supplementary material for: Transmission of extended spectrum β-lactamase-producing Escherichia coli and antimicrobial resistance gene flow across One Health compartments in eastern Africa: a whole-genome sequence analysis from a prospective cohort study
Source: Lancet Microbe. 2026 Jan;7(1):None. doi: 10.1016/j.lanmic.2025.101224 (PMC12888559; doi:10.1016/j.lanmic.2025.101224)
Supplement: Supplementary appendix 1 [file mmc1.pdf]

THE LANCET
Microbe

Supplementary appendix 1

This appendix formed part of the original submission and has been peer reviewed.
We post it as supplied by the authors.

Supplement to: Musicha P, Beale MA, Cocker D, et al. Transmission of extended spectrum β-lactamase-producing *Escherichia coli* and antimicrobial resistance gene flow across One Health compartments in eastern Africa: a whole-genome sequence analysis from a prospective cohort study. *Lancet Microbe* 2025. https://doi.org/10.1016/j.lanmic.2025.101224

Appendix 1 Supplementary methods, results and figures for "Transmission of ESBL producing *E. coli* and antimicrobial resistance gene flow across One Health compartments in Eastern Africa: A whole genome sequencing study"

Patrick Musicha PhD, Mathew A Beale PhD, Derek Cocker PhD, Fiona A Oruru, Allan Zuza, Chifundo Salifu MSc, George Katende, Sylvia Nanono, Fred Isaasi, Kondwani Chidziwisano, Lawrence Mugisha PhD, Henry Kajumbula PhD, David Musoke PhD, Professor Tracy Morse PhD, Shevin Jacob PhD, Professor Nicholas A Feasey PhD, Professor Nicholas R Thomson PhD.

Table of Contents

Appendix 1 Supplementary methods, results and figures for "Transmission of ESBL producing E. coli and antimicrobial resistance gene flow across One Health compartments in Eastern Africa: A whole genome sequencing study" **.................. 1**

Supplementary Methods 2
Study setting and sample collection 2
Microbiology procedures 3
Detailed description of DNA extraction, library preparation and sequencing 3
De novo assembly and sequence annotation 3
Multi-locus sequence typing and characterisation of AMR determinants and plasmids 4
Core genome phylogeny reconstruction and population structure analysis 4
Lineage specific analyses 4
SNP Analysis 5
Statistics 6

Supplementary results 6
Fine scale within ST diversity and phylogenomic analysis 6

Supplementary figures 7

Supplementary tables 14

References 16

Supplementary Methods

Study setting and sample collection

Study setting and sampling strategy are described in more detail elsewhere.[1,2] In summary, the DRUM household study was carried out in Malawi from April 29, 2019 to December 3, 2020 and in Uganda from July 16, 2020 to August,6 2020 to 2021. In Malawi, the study took place in Blantyre (Ndirande and Chileka) and Chikwawa, with site selection based on community acceptance and existing research infrastructure. Ndirande is large high-density urban settlement within Blantyre, while Chileka is peri-urban on the northern outskirts of Blantyre city. The size of the recruitment area was $3km^2$ for Ndirande and $14km^2$ for Chileka. Chikwawa on the other hand, is a large district situated in the southern Shire valley and its northern border is 50 km from Blantyre. It is a rural area, including a mixture of subsistence and large-scale sugar farming, and given its low-lying situation is historically prone to flooding. The study area for Chikwawa was $71km^2$.

In Uganda, research was conducted in Kampala and Hoima, with an aim to include diverse socioeconomic settings. Kampala's sampling area consisted of three adjacent, wedge-shaped polygons (ranging from 1 km^2 to 3.4 km^2), categorised by local socioeconomic status. Hoima, a smaller urban centre in western Uganda, included two separate polygons (3.6 km^2 and 7.6 km^2), representing peri-urban and rural environments, respectively, the later with more human and animal cohabitation.

Samples included in the study were collected from two hundred fity-nine households enrolled at baseline and followed up at one month, three months and six months in Malawi; and an additional ninety-two households enrolled at baseline and followed up at one month, two months and four months in Uganda. On each household visit, samples collected included human and animal stool, environmental samples from specimen such as food, water, door handles and clothes, inside or surrounding the dwelling property of the household, and broader environmental samples such as river water. Data collected at each household visit included individual and household demographics, antimicrobial use, health seeking behaviour and WASH behavioural practices.

Microbiology procedures

All samples were initially inoculated into enrichment broth (Buffered Peptone Water-BPW) and then placed in an aerobic incubator at 37 ± 1˚C for 18–24 hours. After incubation a 1.8 ml aliquot of the culture BPW was stored at -80°C, and a 1μl loop of the remaining sample plated onto ESBL chromogenic agar (CHROMagar™, Saint-Denis, France). Plates were placed in aerobic incubator at 37 ± 1 °C for 18–24 hrs and then read for growth of ESBL bacteria. Pink colonies and (indole positive) white colonies were categorised as ESBL-Ec.[1]

Detailed description of DNA extraction, library preparation and sequencing

DNA was extracted from all ESBL-positive single colony isolates using the QIASymphony DSP Virus/Pathogen mini-kit® on the QIASymphony® (QIAGEN, Hilden, Germany) automated DNA extraction platform or manually extracted using the DNeasy® blood and tissue kit (QIAGEN, Hilden, Germany) at Malawi-Liverpool-Wellcome Programme (MLW), Malawi and the Department of Medical Microbiology, Makere University, Uganda. DNA extracts were shipped to Wellcome Sanger Institute (WSI) where DNA libraries were constructed using NEB Ultra II custom kit on an Agilent Bravo WS automation system and sequenced on the Illumina HiSeq X10 platform (Illumina Inc, San Diego, California, USA) to produce paired-end raw reads of 150 base pairs (bp). Quality of raw reads was assessed using FastQC (https://www.bioinformatics.babraham.ac.uk/projects/fastqc/) and MultiQC.[3] We used Kraken v0.10.6 to confirm sample species and excluded genomes with < 40% of raw reads belonging to *E. coli*.[4] Raw sequence data were deposited in the European Nucleotide Archive (ENA) and ENA accession numbers are included in Table S1.

For 38 selected samples identified as representative of major lineages or MLSTs, we reextracted DNA from the colony isolate using the MasterPure kit (BioSearch Technologies, Hoddesdon, United Kingdom) and performed long-read sequencing on Sequel II (Pacific Biosciences, Menlo Park, California, USA).

De novo assembly and sequence annotation

We ran an WSI automated pipeline to assemble raw reads into contiguous sequences

(contigs) using Spades (v3.14.0) and annotated the assemblies using PROKKA (v1.14.5).[5–7] Quality assessment of genome sequence assemblies was performed as follows: we filtered out contigs of length <300 bp; generated assembly statistics and excluded from further analysis assemblies with total size < 4 mega base pairs (MB) or > 6MB or with N50 < 80,000 bp.[8] We assessed genome completeness and contamination using checkM (v1.2.2); and excluded genomes with <90% completeness or > 5% contamination.[9]

Multi-locus sequence typing and characterisation of AMR determinants and plasmids

Multi-locus sequence typing (MLST) was performed *in silico* using mlst tool (v2.16.2) (https://github.com/tseemann/mlst). We screened for AMR determinants using AMRfinder plus (v3.10.40) and used the mob-recon function in mob-suite (v3.0.3) to identify and cluster plasmid contigs and type them incompatibility groups.[10,11] Where relevant, we used minimum thresholds of 95% blastp identity and 90% coverage.

Core genome phylogeny reconstruction and population structure analysis

We inferred a pan-genome using the default settings of panaroo (v1.3.0) and classified genes identified in at least 99% of the genomes as core.[12] We concatenated alignments of the core genes to generate a core gene alignment. Single nucleotide polymorphic (SNP) sites were extracted from the core gene alignment using snp-sites (v2.5.1) to generate a core-SNP alignment.[13] A core gene maximum likelihood (ML) phylogenetic tree was constructed from the core SNP alignment using IQ-TREE v1.6.12 GTR+I+G model.[14] Reliability of the inferred branches and branch partitions in the phylogenetic tree was assessed with 1000 ultrafast bootstrap replicates using UFBoot2.[15] Phylogenetic trees were visualised and annotated using ggtree (v3.5.1) package in R (v4.2.1) and iTOL.[16–18] We performed population structure analysis on the whole collection using PopPUNK (v2.6) and assigned lineages based on a previously described PopPUNK reference database for *E. coli.*[19,20]

Lineage specific analyses

We performed lineage specific analyses on the five most common STs in this collection (ST131, ST38, ST10, ST617 and ST3580), each comprising 100 or more genomes. We randomly selected 2-6 isolates from each of these five STs and performed long-read sequencing using

the Pacific Biosciences (PacBio) platform to generate high quality reference genomes that were phylogenetically representative of the major lineages in our collection. We assembled *de novo* the resulting long-read raw data with flye (v2.9.2) assembler and polished the assemblies with their corresponding Illumina short-reads using polypolish(v0.5.0).[21,22] We used Quast (v5.0.2) to assess the quality of the generated hybrid assemblies.[23] For each of the four STs, the hybrid assembly with the highest quality scores was selected as the local ST-specific reference genome, to which all short reads of genomes in that ST were mapped using BWA aligner followed by INDEL realignment using GATK (v3.4.46) Indel Realigner.[24,25] Variant calling and consensus pseudosequence generation were performed using samtools (v1.2) and bcftools (v1.2). A minimum of 8 supporting reads (3 per strand) and a variant frequency/mapping quality cut-off of 0.8 were used to call variants. Sites not meeting these criteria were masked to 'N' in the pseudosequence. We used Gubbins (v3.2.1) to identify and mask recombination sites and constructed recombination-free phylogenetic trees with IQTree.[26] We performed ST-specific population structure analyses using RhierBAPS (v1.1.4).[27] [28]

SNP Analysis

For ST-specific multiple sequence alignments (for ST10, ST38, ST131, ST167), we inferred pairwise SNP distances using pairsnp (v0.1.0) available at https://github.com/gtonkinhill/pairsnp). We linked metadata to samples in R and evaluated the distribution of pairwise SNP distances for each ST sequence alignment within and between countries, households, and individual people, examining SNP distributions to determine a threshold for including the 5% closest comparisons (≤2 SNPs in all cases). We inferred putative transmission networks by constructing edge-networks from all pairwise comparisons below a series of thresholds (0, 1, 2, 5, 10 and 20 SNPs) using the network v1.18.1 and iGraph (v1.5.0) packages, and these were plotted using ggnetwork (v0.5.12) in R[29–31]. We quantified putative transmission events at each threshold by counting the number of pairwise comparisons between each sample source type (e.g. human, environment, animal). Where multiple samples from each human individual were present, we deduplicated pairwise comparisons less than or equal to the threshold being evaluated for transmission.

Statistics

Associations between ST versus country and ST versus ecological compartment were performed using chi-square test or Fisher's exact test where appropriate. We performed a multinomial regression to quantify the association between plasmid cluster distribution and and sample country of origin and ecological source. We considered Y_i to be the outcome variable which could take J discrete possible outcomes {1, 2, …, J}. We let π_{ij} be the probability that outcome Y_i associated with sample i, will take the value j (i.e. $\pi_{ij} = \Pr\,(Y_i = j)$. Thus, the data generating process of Y_i follows the multinomial distribution $Y_i \sim multinomial(Y_i|\pi_{ik})$, for $k = 1,2, \ldots, J$ and $\sum_{j=1}^{J} \pi_j = 1$. Given a vector of covariates $\boldsymbol{X} = (x_1, x_2, \ldots, x_D)$,

$$Pr(Y_i = j|X_i) = \pi_{ij}(X_i) = \frac{\exp\,(\alpha_j + \beta_{1i}x_1 + \cdots + \beta_D x_D)}{\sum_{k=1}^{J} \exp\,(\alpha_k + \beta_1 x_1 + \cdots + \beta_D x_D)}$$

Here, our response variable **Y** is ST and the set of covariates $\boldsymbol{X} = (x_1 = country, x_2 = ecological\ source)$. We used the nnet package in R to implement the multinomial regression.

Supplementary results

Fine scale within ST diversity and phylogenomic analysis

Hierarchical Bayesian Analysis of Population Structure (hierBAPS) was used to subdivide the STs into hierBAPS lineages (Figure 2). ST10 isolates were subdivided into five hierBAPS level1 lineages. The largest ST10 lineage (lineage 3) consisted of closely related genomes predominantly from Malawi. The remaining four ST10 lineages consisted of less related genomes, from both Malawi and Uganda, that were characterised by deep-rooted branches (Figure 2A). ST131 and ST167 were each delineated into four hierBAPS level 1 lineages and ST38 into five lineages (Figure 2A-D). ST3580, which was separated into three hierBAPS lineages, was the most clonal among the five major STs, with the two most divergent genomes having a genetic distance of 94 SNPs (Figure 2E). ST10 was the most diverse ST amongst the major five STs, with ST131, ST167 and ST38 and 3580 comprising of more closely related genomes characterised by relatively shorter branch lengths than ST10. Across all the five STs,

there was strong phylogenetic mixing of isolates from all the three ecological compartments, but although interspersing of isolates from the two countries could be observed, we also found phylogenetic clustering of genomes by country of origin (Figure 2).

Supplementary figures

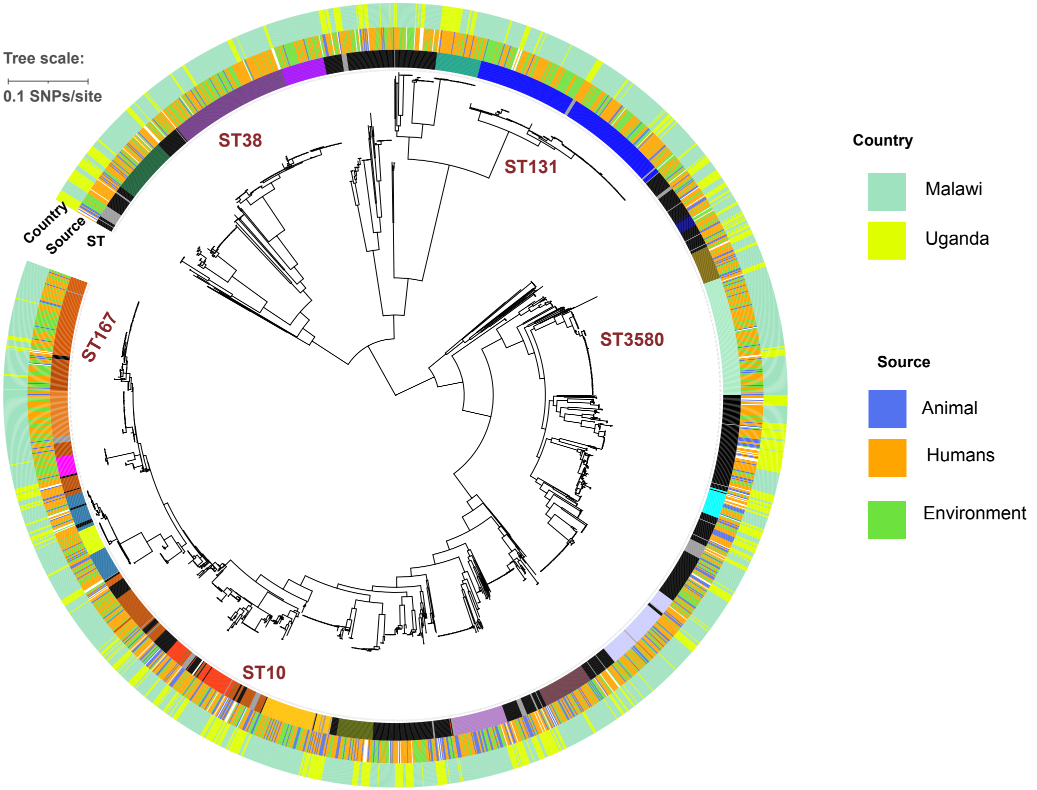

Figure S1: *Maximum likelihood core-genome phylogenetic tree of ESBL producing carriage E. coli from Uganda and Malawi. The phylogenetic tree is mid-point rooted. The inner ring shows sequence type distribution across the tree, the middle ring shows isolate ecological source as human stool, animal stool or the environment and the outer ring shows isolate country of origin, either Malawi or Uganda.*

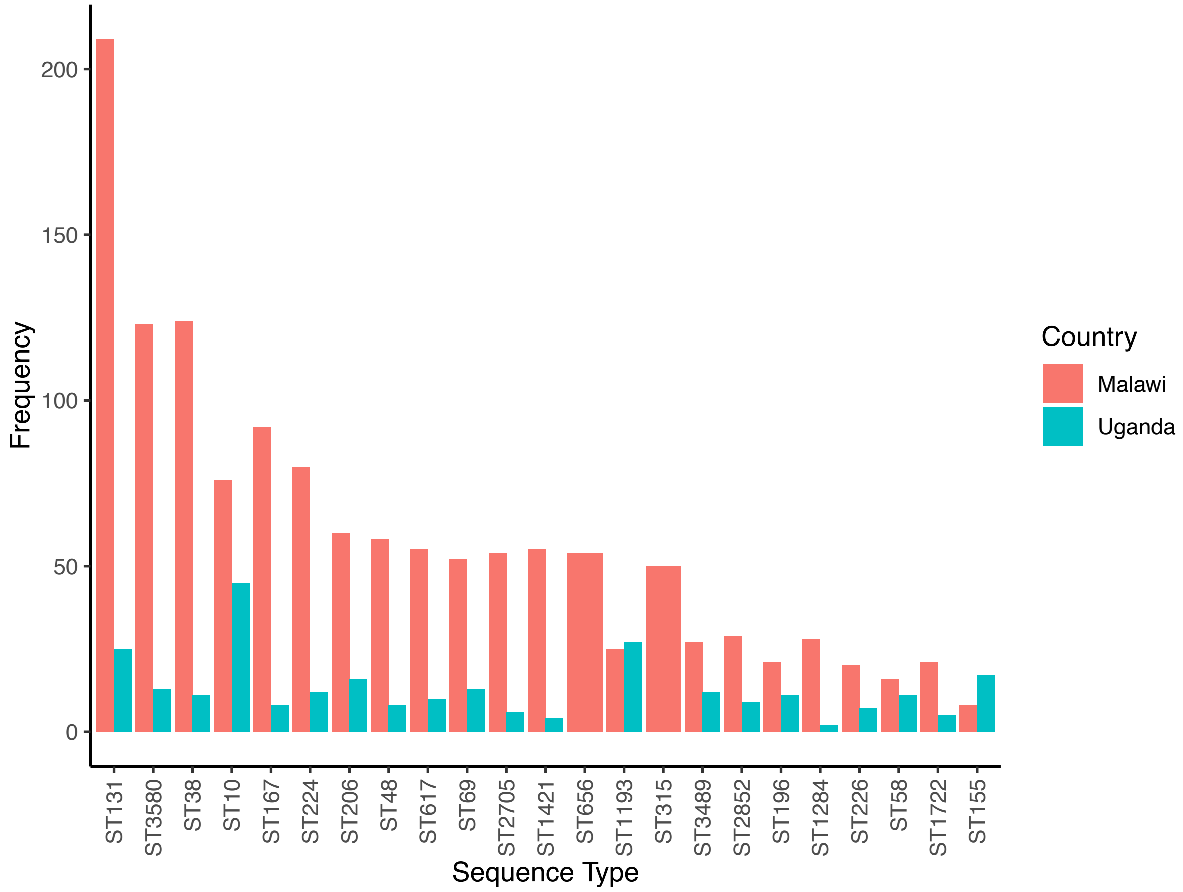

Figure S2: *Distribution of sequence types with at least 1% of genomes, by sample country of origin*

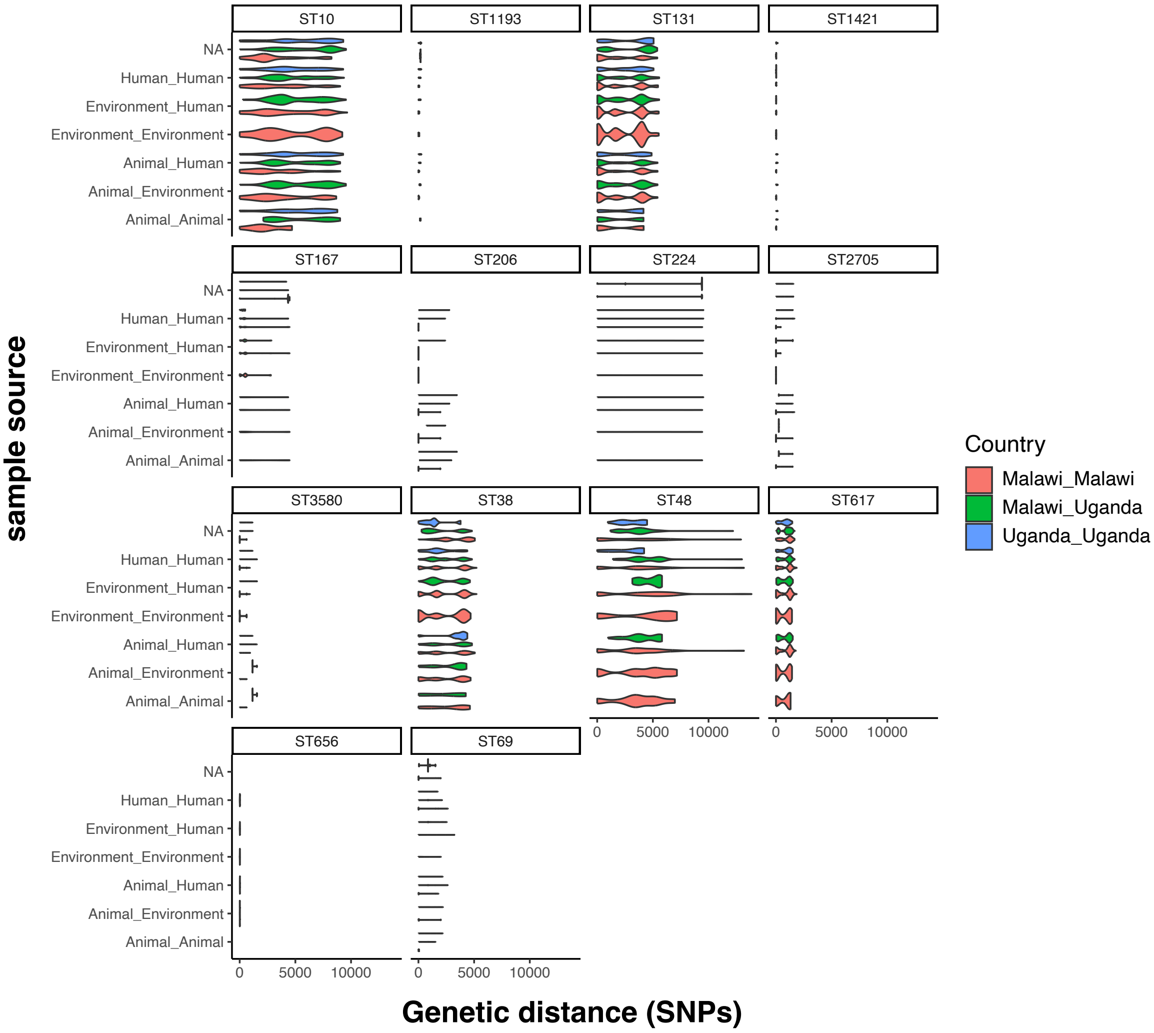

Figure S3: *Distribution of core genome SNP distances within sequence types with > 50 genomes.*

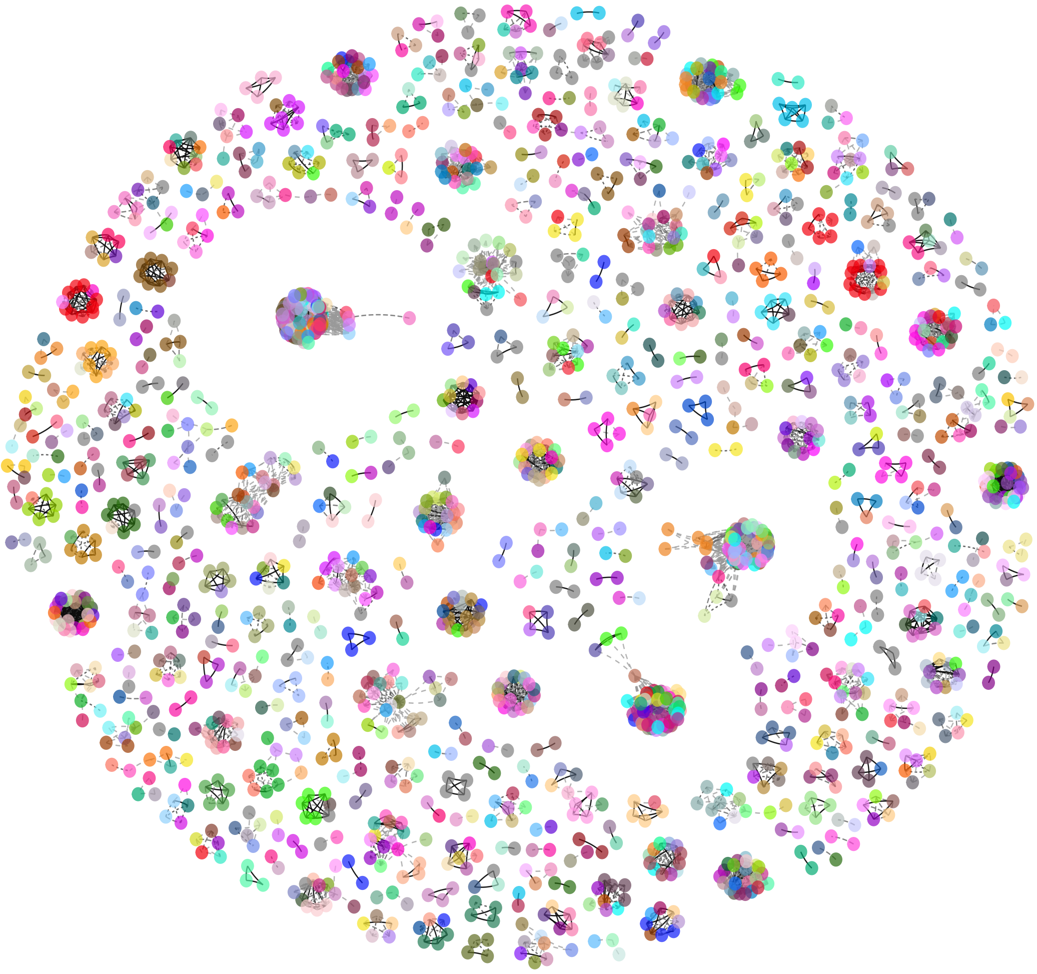

***Figure S4**: ESBL E. coli Transmission cluster-household linkage. A core genome pairwise SNP distance-based transmission network for all ESBL E. coli genomes in the DRUM collection with ≤ 5 pairwise SNP distances coloured. Each network node represents a genome and is coloured by the sample household ID.*

Extended data.

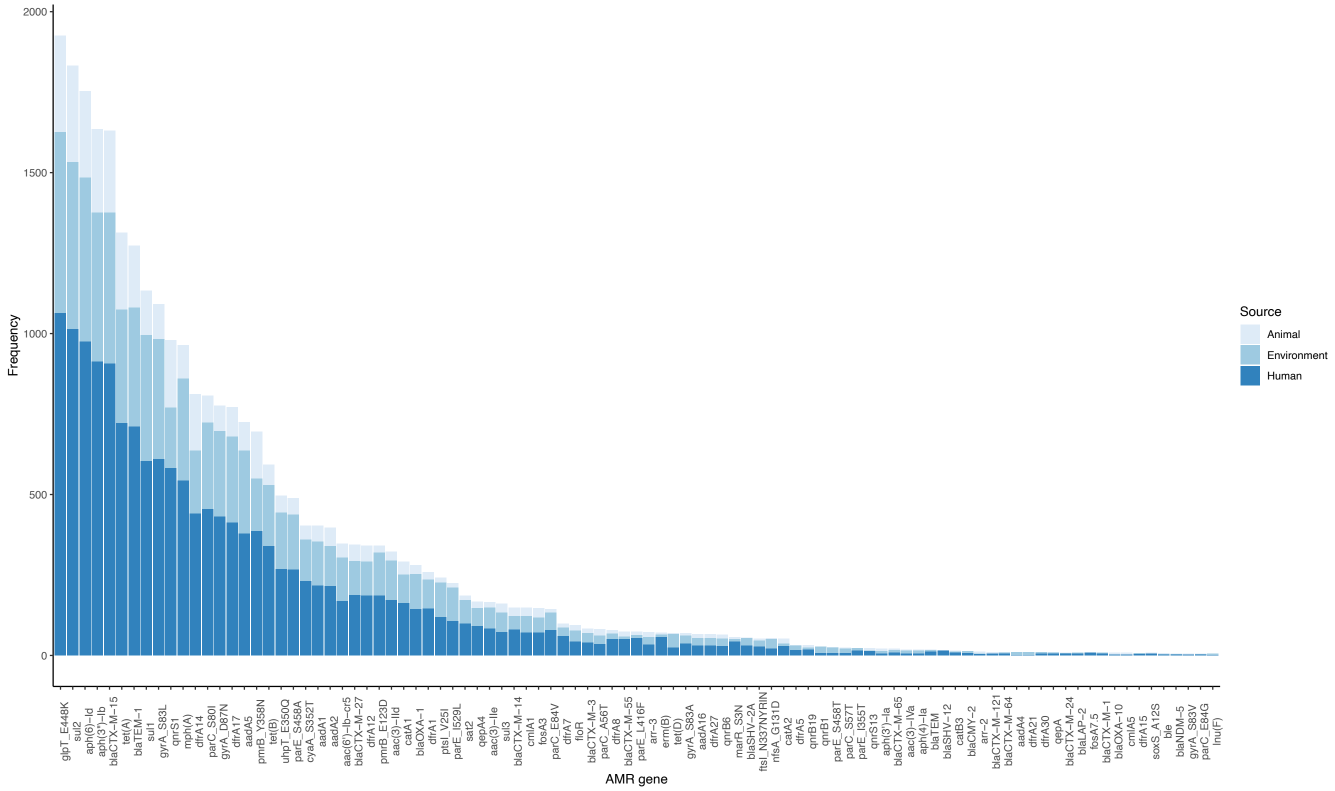

Figure S5: *Distribution of AMR genes by ecological compartment. Figure only shows AMR genes present in at least five genomes.*

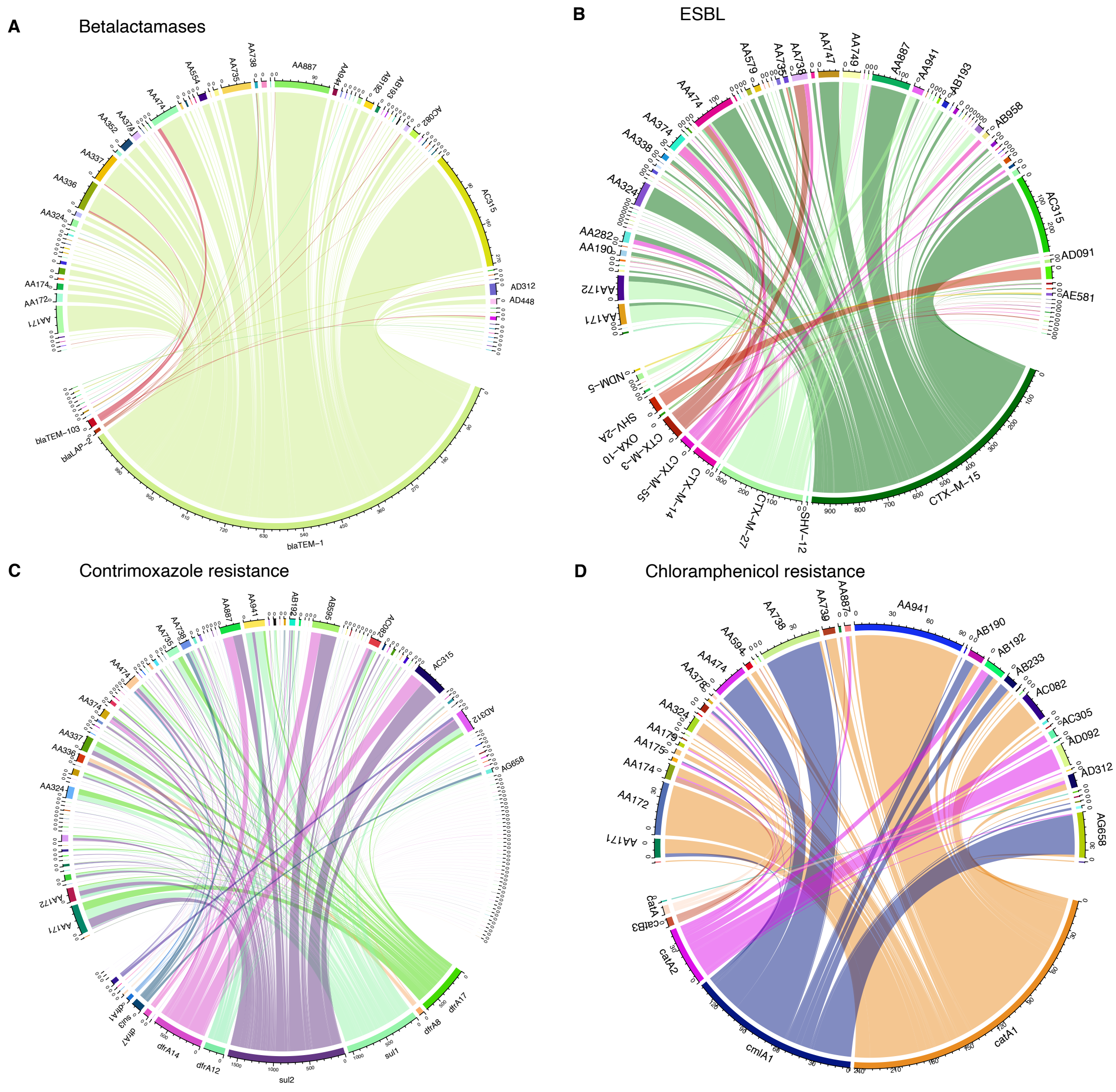

__Figure S6:__ Circos plots showing links between plasmids clusters and genes they carry encoding resistance to (a) narrow spectrum beta-lactams (b) 3rd generation cephalosporins (extended spectrum beta-lactams (ESBLs)) (c) cotrimoxazole and (d) chloramphenicol .

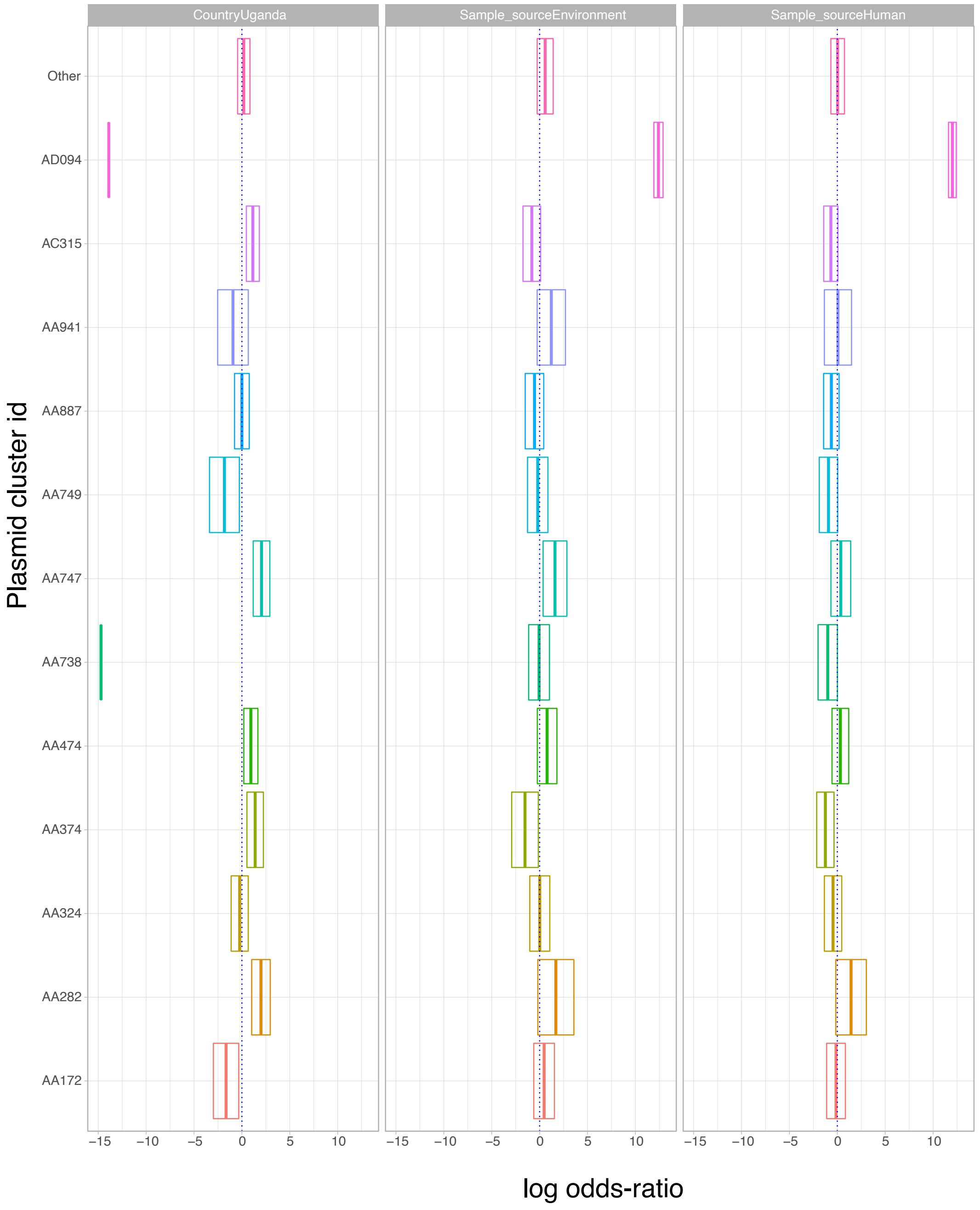

Figure S7*: Association between ESBL plasmid and sample country or ecological source. Associations are presented as cross bars showing odds ratio (on a log scale) of a plasmid cluster being associated with a particular country (Uganda relative to Malawi) or ecological source (environment or human relative to animal). The log-odd ratio values in shown by the middle vertical line of the cross bar and boundaries of the cross bar on the left and right of the odds-ratio line represent the lower and upper bounds of a 95% confidence interval.*

A

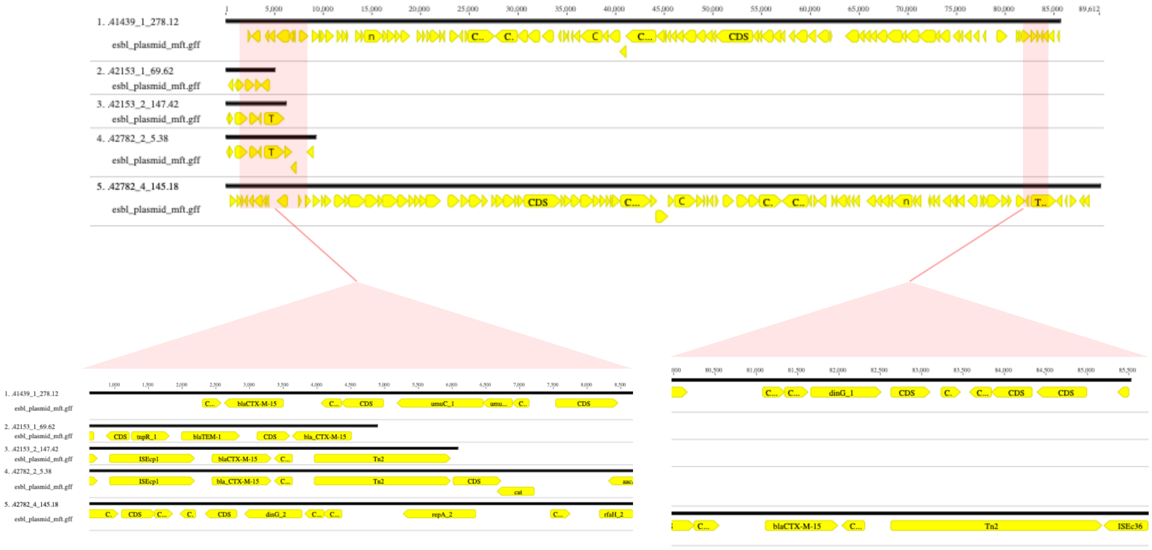

B

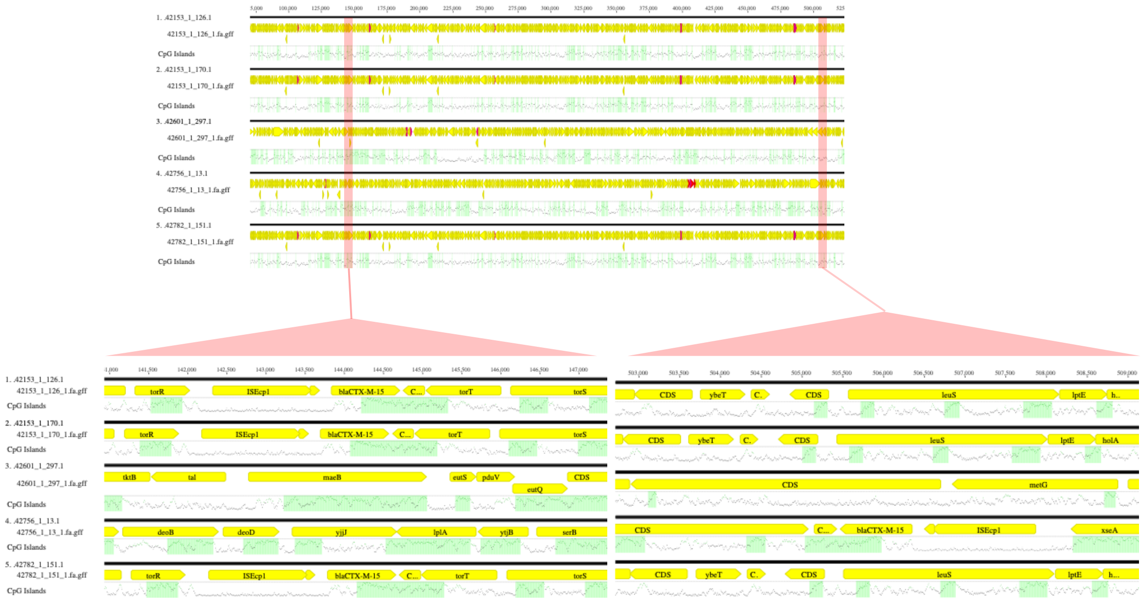

***Figure S8**: Genetic environments of ESBL genes (A) on plasmid and (B) on chromosome sequences*

Supplementary tables

Supplementary table 1: List of isolates submitted to Enterobase for multi-locus sequencing typing and their identified sequence types

Sanger_lane_	Date Entered	Release Date	ST	ST Complex	Lineage	adk	fumC	gyrB	icd	mdh	purA	recA
41439_143	13/12/2023	13/12/2023	7696	ST10 Cplx	A	744	11	4	8	8	8	2
42601_1_342	14/12/2023	14/12/2023	8025	ST10 Cplx	A	10	7	4	8	8	5	2
42782_1_53	08/01/2024	08/01/2024	8025	ST10 Cplx	A	10	7	4	8	8	5	2
42782_1_147	08/01/2024	08/01/2024	8025	ST10 Cplx	A	10	7	4	8	8	5	2
42782_1_157	08/01/2024	08/01/2024	8025	ST10 Cplx	A	10	7	4	8	8	5	2
42782_1_303	08/01/2024	08/01/2024	8025	ST10 Cplx	A	10	7	4	8	8	5	2
42782_1_328	08/01/2024	08/01/2024	8025	ST10 Cplx	A	10	7	4	8	8	5	2
42782_2_14	08/01/2024	08/01/2024	8025	ST10 Cplx	A	10	7	4	8	8	5	2
41439_1_288	13/12/2023	13/12/2023	8986	ST10 Cplx	A	10	7	4	8	12	109	2
42756_4_180	15/12/2023	15/12/2023	8986	ST10 Cplx	A	10	7	4	8	12	109	2
41330_8_58	13/12/2023	13/12/2023	9525	ST10 Cplx	A	10	11	4	140	8	1	2
41330_8_51	13/12/2023	13/12/2023	13391	ST10 Cplx	A	10	11	1209	8	8	8	2
41439_1_268	13/12/2023	13/12/2023	13391	ST10 Cplx	A	10	11	1209	8	8	8	2
42756_3_126	15/12/2023	15/12/2023	13391	ST10 Cplx	A	10	11	1209	8	8	8	2
42782_4_291	10/01/2024	10/01/2024	13814	ST10 Cplx		6	11	4	1	8	8	133
42756_3_90	15/12/2023	15/12/2023	14131	ST10 Cplx	A	10	11	4	8	8	1183	73
41439_1_13	13/12/2023	13/12/2023	14381	ST10 Cplx		6	11	1441	8	8	8	2
41439_1_24	13/12/2023	13/12/2023	15381	ST10 Cplx	A	6	2452	4	8	8	8	2
41439_1_29	13/12/2023	13/12/2023	15381	ST10 Cplx	A	6	2452	4	8	8	8	2
41439_1_65	13/12/2023	13/12/2023	15381	ST10 Cplx	A	6	2452	4	8	8	8	2
41439_1_116	13/12/2023	13/12/2023	15381	ST10 Cplx	A	6	2452	4	8	8	8	2
41439_1_164	13/12/2023	13/12/2023	15381	ST10 Cplx	A	6	2452	4	8	8	8	2
41439_1_113	13/12/2023	13/12/2023	15384	ST10 Cplx	A	10	11	4	8	1511	8	2
42153_1_62	13/12/2023	13/12/2023	15386	ST10 Cplx	A	10	11	4	8	10	13	2
42782_1_334	08/01/2024	08/01/2024	15466	ST10 Cplx	A	30	11	5	8	7	1200	2
42782_2_57	08/01/2024	08/01/2024	15467	ST10 Cplx	A	1294	11	4	8	8	13	2
42782_4_298	10/01/2024	10/01/2024	15475	ST10 Cplx	A	6	11	4	8	8	8	1221
43416_1_222	11/01/2024	11/01/2024	15486	ST10 Cplx	A	10	11	4	1954	8	8	2
42153_2_240	14/12/2023	14/12/2023	11380	ST131 Cplx	B2	53	40	47	1391	36	28	29
42153_2_298	14/12/2023	14/12/2023	11380	ST131 Cplx	B2	53	40	47	1391	36	28	29
42782_4_354	10/01/2024	10/01/2024	11380	ST131 Cplx	B2	53	40	47	1391	36	28	29
41330_8_52	13/12/2023	13/12/2023	15379	ST131 Cplx	B2	53	40	47	13	1509	28	29
41439_1_248	13/12/2023	13/12/2023	15379	ST131 Cplx	B2	53	40	47	13	1509	28	29
42756_3_213	15/12/2023	15/12/2023	15379	ST131 Cplx	B2	53	40	47	13	1509	28	29
43416_1_30	11/01/2024	11/01/2024	15379	ST131 Cplx	B2	53	40	47	13	1509	28	29
42782_2_276	10/01/2024	10/01/2024	7322	ST155 Cplx		6	1007	14	16	24	8	14
42782_2_42	08/01/2024	08/01/2024	8679	ST155 Cplx		6	4	14	16	748	8	14
41439_1_255	13/12/2023	13/12/2023	8744	ST155 Cplx		6	4	14	989	24	8	14
42756_3_19	14/12/2023	14/12/2023	8744	ST155 Cplx		6	4	14	989	24	8	14
42756_3_57	14/12/2023	14/12/2023	8744	ST155 Cplx		6	4	14	989	24	8	14
42756_3_148	15/12/2023	15/12/2023	8744	ST155 Cplx		6	4	14	989	24	8	14
42153_1_78	14/12/2023	14/12/2023	11189	ST155 Cplx		6	1181	14	16	24	8	14
42153_1_245	14/12/2023	14/12/2023	11189	ST155 Cplx		6	1181	14	16	24	8	14
42153_1_275	14/12/2023	14/12/2023	11189	ST155 Cplx		6	1181	14	16	24	8	14
42153_2_341	14/12/2023	14/12/2023	11189	ST155 Cplx		6	1181	14	16	24	8	14
42601_1_320	14/12/2023	14/12/2023	11189	ST155 Cplx		6	1181	14	16	24	8	14
42782_1_32	08/01/2024	08/01/2024	11189	ST155 Cplx		6	1181	14	16	24	8	14
42782_1_210	08/01/2024	08/01/2024	11189	ST155 Cplx		6	1181	14	16	24	8	14
42782_2_101	08/01/2024	08/01/2024	11189	ST155 Cplx		6	1181	14	16	24	8	14
42782_2_109	08/01/2024	08/01/2024	11189	ST155 Cplx		6	1181	14	16	24	8	14
42782_2_116	08/01/2024	08/01/2024	11189	ST155 Cplx		6	1181	14	16	24	8	14
42782_2_119	08/01/2024	08/01/2024	11189	ST155 Cplx		6	1181	14	16	24	8	14
42782_2_215	08/01/2024	08/01/2024	11189	ST155 Cplx		6	1181	14	16	24	8	14
42782_4_217	10/01/2024	10/01/2024	15472	ST155 Cplx		6	2465	14	16	24	8	14
41439_1_36	13/12/2023	13/12/2023	15383	ST156 Cplx	AxB1	1861	29	32	16	11	8	44
42153_2_294	14/12/2023	14/12/2023	9816	ST206 Cplx	AxB1	6	7	856	1	8	18	2
41439_1_104	13/12/2023	13/12/2023	10822	ST206 Cplx	AxB1	1110	7	5	1	8	18	2
42756_3_1	14/12/2023	14/12/2023	10822	ST206 Cplx	AxB1	1110	7	5	1	8	18	2
42756_4_183	15/12/2023	15/12/2023	9347	ST23 Cplx		6	4	14	1084	20	62	7
43416_1_192	11/01/2024	11/01/2024	15487	ST23 Cplx	B1	6	4	33	132	20	12	949
42782_1_322	08/01/2024	08/01/2024	3385	ST38 Cplx	D	4	-43	2	25	5	5	19
42601_1_242	14/12/2023	14/12/2023	15389	ST38 Cplx	D	271	26	39	25	1513	31	19
42756_4_115	15/12/2023	15/12/2023	15401	ST394 Cplx	D	21	35	1539	52	5	5	4
43416_1_102	11/01/2024	11/01/2024	15401	ST394 Cplx	D	21	35	1539	52	5	5	4
41439_1_250	13/12/2023	13/12/2023	12637	ST469 Cplx	AxB1	109	65	244	1	9	13	14
41330_8_224	13/12/2023	13/12/2023	8881	ST648 Cplx		92	4	87	96	70	13	2
41439_1_213	13/12/2023	13/12/2023	8881	ST648 Cplx		92	4	87	96	70	13	2
42476_3_9	14/12/2023	14/12/2023	8881	ST648 Cplx		92	4	87	96	70	13	2
42756_3_261	15/12/2023	15/12/2023	8881	ST648 Cplx		92	4	87	96	70	13	2
43416_1_173	11/01/2024	11/01/2024	8881	ST648 Cplx		92	4	87	96	70	13	2
43416_1_238	11/01/2024	11/01/2024	8881	ST648 Cplx		92	4	87	96	70	13	2
42756_3_40	14/12/2023	14/12/2023	7937			6	4	15	16	11	8	6
41439_1_154	13/12/2023	13/12/2023	8131		D	5	3	2	6	45	5	4
43416_1_41	11/01/2024	11/01/2024	8131		D	5	3	2	6	45	5	4
42756_3_36	14/12/2023	14/12/2023	8330			806	1096	701	520	401	40	350
42756_3_153	15/12/2023	15/12/2023	8577		A	8	7	728	220	8	8	2
43416_1_78	11/01/2024	11/01/2024	8577		A	8	7	728	220	8	8	2
41330_8_277	13/12/2023	13/12/2023	9439		A	10	11	1	8	12	18	2
41430_160	13/12/2023	13/12/2023	9523		ABD	12	371	176	12	1	2	2
41439_1_267	13/12/2023	13/12/2023	9523		ABD	12	371	176	12	1	2	2
43416_1_20	11/01/2024	11/01/2024	9523		ABD	12	371	176	12	1	2	2
41330_8_185	13/12/2023	13/12/2023	10391			218	371	53	140	247	2	216
43416_1_235	11/01/2024	11/01/2024	12137			6	4	1159	102	9	73	682
42782_3_163	10/01/2024	10/01/2024	12290		ABD	826	186	54	10	1	35	47
41439_1_11	13/12/2023	13/12/2023	12400		A	8	1873	1	8	8	18	6
42153_1_254	14/12/2023	14/12/2023	12569		D	200	3	174	6	1187	5	191
42601_1_214	14/12/2023	14/12/2023	12569		D	200	3	174	6	1187	5	191
42782_1_67	08/01/2024	08/01/2024	12569		D	200	3	174	6	1187	5	191
42782_1_115	08/01/2024	08/01/2024	12569		D	200	3	174	6	1187	5	191
42782_1_179	08/01/2024	08/01/2024	12569		D	200	3	174	6	1187	5	191
42782_3_52	09/01/2024	09/01/2024	12569		D	200	3	174	6	1187	5	191
42782_3_72	09/01/2024	09/01/2024	12569		D	200	3	174	6	1187	5	191
42782_3_104	10/01/2024	10/01/2024	12569		D	200	3	174	6	1187	5	191
42782_3_211	10/01/2024	10/01/2024	12569		D	200	3	174	6	1187	5	191
42782_3_303	10/01/2024	10/01/2024	12569		D	200	3	174	6	1187	5	191

References

1. Cocker D, Sammarro M, Chidziwisano K, *et al.* Drivers of Resistance in Uganda and Malawi (DRUM): a protocol for the evaluation of One-Health drivers of Extended Spectrum Beta Lactamase (ESBL) resistance in Low-Middle Income Countries (LMICs). *Wellcome Open Res* 2022; **7**: 55.
2. Cocker D, Chidziwisano K, Mphasa M, *et al.* Investigating One Health risks for human colonisation with extended spectrum β-lactamase-producing Escherichia coli and Klebsiella pneumoniae in Malawian households: a longitudinal cohort study. *Lancet Microbe* 2023; **4**: e534–43.
3. Ewels P, Magnusson M, Lundin S, Käller M. MultiQC: Summarize analysis results for multiple tools and samples in a single report. *Bioinformatics* 2016; **32**: 3047–8.
4. Wood DE, Salzberg SL. Kraken: ultrafast metagenomic sequence classification using exact alignments. 2014 http://ccb.jhu.edu/software/kraken/.
5. Prjibelski A, Antipov D, Meleshko D, Lapidus A, Korobeynikov A. Using SPAdes De Novo Assembler. *Curr Protoc Bioinformatics* 2020; **70**: e102.
6. Seemann T. Prokka: Rapid prokaryotic genome annotation. *Bioinformatics* 2014; **30**: 2068–9.
7. Page AJ, De Silva N, Hunt M, *et al.* Robust high-throughput prokaryote de novo assembly and improvement pipeline for Illumina data. *Microb Genom* 2016; **2**: e000083.
8. Sørensen LH, Pedersen SK, Jensen JD, *et al.* Whole-genome sequencing for antimicrobial surveillance: species-specific quality thresholds and data evaluation from the network of the European Union Reference Laboratory for Antimicrobial Resistance genomic proficiency tests of 2021 and 2022. *mSystems* 2024; **9**. DOI:10.1128/msystems.00160-24.
9. Parks DH, Imelfort M, Skennerton CT, Hugenholtz P, Tyson GW. CheckM: Assessing the quality of microbial genomes recovered from isolates, single cells, and metagenomes. *Genome Res* 2015; **25**: 1043–55.
10. Feldgarden M, Brover V, Gonzalez-Escalona N, *et al.* AMRFinderPlus and the Reference Gene Catalog facilitate examination of the genomic links among antimicrobial resistance, stress response, and virulence. *Sci Rep* 2021; **11**. DOI:10.1038/s41598-021-91456-0.
11. Robertson J, Nash JHE. MOB-suite: software tools for clustering, reconstruction and typing of plasmids from draft assemblies. DOI:10.6084/m9.figshare.6177188.
12. Tonkin-Hill G, MacAlasdair N, Ruis C, *et al.* Producing polished prokaryotic pangenomes with the Panaroo pipeline. *Genome Biol* 2020; **21**. DOI:10.1186/s13059-020-02090-4.
13. Page AJ, Taylor B, Delaney AJ, *et al.* SNP-sites: rapid efficient extraction of SNPs from multi-FASTA alignments. *Microb Genom* 2016; **2**: e000056.
14. Minh BQ, Schmidt HA, Chernomor O, *et al.* IQ-TREE 2: New Models and Efficient Methods for Phylogenetic Inference in the Genomic Era. *Mol Biol Evol* 2020; **37**: 1530–4.

15 Thi Hoang D, Chernomor O, von Haeseler A, Quang Minh B, Sy Vinh L, Rosenberg MS. UFBoot2: Improving the Ultrafast Bootstrap Approximation. *Mol Biol Evol* 2017; **35**: 518–22.
16 Xu S, Li L, Luo X, *et al.* Ggtree: A serialized data object for visualization of a phylogenetic tree and annotation data. *iMeta* 2022; **1**. DOI:10.1002/imt2.56.
17 R Core Team. R: A language and environment for statistical computing. 2022.
18 Letunic I, Bork P. Interactive tree of life (iTOL) v5: An online tool for phylogenetic tree display and annotation. *Nucleic Acids Res* 2021; **49**: W293–6.
19 Lees JA, Harris SR, Tonkin-Hill G, *et al.* Fast and flexible bacterial genomic epidemiology with PopPUNK. *Genome Res* 2019; **29**: 304–16.
20 Horesh G, Blackwell GA, Tonkin-Hill G, Corander J, Heinz E, Thomson NR. A comprehensive and high-quality collection of escherichia coli genomes and their genes. *Microb Genom* 2021; **7**: 1–15.
21 Kolmogorov M, Yuan J, Lin Y, Pevzner PA. Assembly of long, error-prone reads using repeat graphs. *Nat Biotechnol* 2019; **37**: 540–6.
22 Wick RR, Holt KE. Polypolish: Short-read polishing of long-read bacterial genome assemblies. *PLoS Comput Biol* 2022; **18**. DOI:10.1371/journal.pcbi.1009802.
23 Mikheenko A, Prjibelski A, Saveliev V, Antipov D, Gurevich A. Versatile genome assembly evaluation with QUAST-LG. In: Bioinformatics. Oxford University Press, 2018: i142–50.
24 Li H. Aligning sequence reads, clone sequences and assembly contigs with BWA-MEM. 2013; published online March 16. http://arxiv.org/abs/1303.3997.
25 McKenna A, Hanna M, Banks E, *et al.* The genome analysis toolkit: A MapReduce framework for analyzing next-generation DNA sequencing data. *Genome Res* 2010; **20**: 1297–303.
26 Croucher NJ, Page AJ, Connor TR, *et al.* Rapid phylogenetic analysis of large samples of recombinant bacterial whole genome sequences using Gubbins. *Nucleic Acids Res* 2015; **43**: e15.
27 Tonkin-Hill G, Lees JA, Bentley SD, Frost SDW, Corander J. RhierBAPs: An R implementation of the population clustering algorithm hierbaps. *Wellcome Open Res* 2018; **3**. DOI:10.12688/wellcomeopenres.14694.1.
28 Petty NK, Zakour NLB, Stanton-Cook M, *et al.* Global dissemination of a multidrug resistant Escherichia coli clone. *Proc Natl Acad Sci U S A* 2014; **111**: 5694–9.
29 Butts CT. Journal of Statistical Software network: A Package for Managing Relational Data in R. 2008 http://www.jstatsoft.org/.
30 Briatte F, Bojanowski M, Canouil M, *et al.* ggnetwork: Geometries to Plot Networks with “ggplot2”. 2023. https://github.com/briatte/ggnetwork (accessed Jan 11, 2024).
31 Csárdi G, Nepusz T. The igraph software package for complex network research. .
